# A common wild rice-derived *BOC1* allele reduces callus browning in *indica* rice transformation

**DOI:** 10.1038/s41467-019-14265-0

**Published:** 2020-01-23

**Authors:** Kun Zhang, Jingjing Su, Min Xu, Zhihui Zhou, Xiaoyang Zhu, Xin Ma, Jingjing Hou, Lubin Tan, Zuofeng Zhu, Hongwei Cai, Fengxia Liu, Hongying Sun, Ping Gu, Chen Li, Yuntao Liang, Wensheng Zhao, Chuanqing Sun, Yongcai Fu

**Affiliations:** 1 MOE Key Laboratory of Crop Heterosis and Utilization, National Center for Evaluation of Agricultural Wild Plants (Rice), Beijing Key Laboratory of Crop Genetic Improvement, Department of Plant Genetics and Breeding, China Agricultural University, Beijing, 100193 China; 2 Rice Research Institute, Guangdong Academy of Agricultural Sciences, Guangzhou, 510640 China; 3 Rice Research Institute, Guangxi Academy of Agricultural Sciences, Nanning, 530007 China; 4 State Key Laboratory of Agrobiotechnology and MOA Key Lab of Pest Monitoring and Green Management, China Agricultural University, Beijing, 100193 China; 5 State Key Laboratory of Plant Physiology and Biochemistry, China Agricultural University, Beijing, 10093 China

**Keywords:** Molecular engineering in plants, Agricultural genetics, Natural variation in plants

## Abstract

Callus browning, a common trait derived from the *indica* rice cultivar (*Oryza sativa* L.), is a challenge to transformation regeneration. Here, we report the map-based cloning of *BROWNING OF CALLUS1* (*BOC1*) using a population derived from crossing Teqing, an elite *indica* subspecies exhibiting callus browning, and Yuanjiang, a common wild rice accession (*Oryza rufipogon* Griff.) that is less susceptible to callus browning. We show that *BOC1* encodes a SIMILAR TO RADICAL-INDUCED CELL DEATH ONE (SRO) protein. Callus browning can be reduced by appropriate upregulation of *BOC1*, which consequently improves the genetic transformation efficiency. The presence of a *Tourist*-like miniature inverted-repeat transposable element (*Tourist* MITE) specific to wild rice in the promoter of *BOC1* increases the expression of *BOC1* in callus. *BOC1* may decrease cell senescence and death caused by oxidative stress. Our study provides a gene target for improving tissue culturability and genetic transformation.

## Abstract (web summary)

Callus browning heavily affects *indica* rice transformation regeneration. Here, the authors show transposon insertion in the promoter of *BOC1* gene, encoding a SIMILAR TO RADICAL-INDUCED CELL DEATH ONE protein, can upregulate its expression and decrease callus browning in cultivated rice by releasing oxidative stress.

## Introduction

In contrast to *japonica* rice (*Oryza sativa* ssp. *japonica*), *Agrobacterium tumefaciens*-mediated transformation of *indica* rice (*Oryza sativa* ssp. *indica*) is hindered by callus browning. Callus browning is a common feature of many plant species that presents problems for in vitro culture, a process required for transgenic breeding, resulting in decreased regenerative ability, poor growth, and even death^1^. The use of antioxidants^2–4^, adsorbing agents^5^, low salt concentrations^6^, and growth regulators^7^ may lessen the effects of callus browning to a certain degree, but because some species do not tolerate these treatments, there is no universal solution to this problem.

The physiological and biochemical mechanisms of callus browning have been extensively studied. For instance, reducing the activity of polyphenol oxidase (PPO), which catalyzes the oxidation of phenols to quinones that in turn polymerize to form brown pigments^8^, can mitigate the browning of callus^9^. Browning calli have lower antioxidant enzyme activity than non-browning calli and oxidative stress is associated with callus browning^10,11^. Endogenous^12^ and exogenous^13^ ethylene (ET) cause browning in plant cell cultures. In addition, reduced nitrite reductase activity leads to callus browning^14^.

Different periods of in vitro rice tissue culture are controlled by many complex genes, respectively^15^. Several quantitative trait loci (QTLs) for culturability traits in rice have been identified^16–24^. Using positional cloning, a major QTL gene encoding ferredoxin-nitrite reductase (NiR) that determines regeneration ability in rice was identified^19^. QTLs involved in callus browning have been identified on all 12 rice chromosomes^17,21–24^. However, to our knowledge, there have been no reports on the isolation of genes related to callus browning in any plant species. The molecular mechanisms underlying physiological processes inducing browning in plant cell culture still remain unclear.

Genetic analyses of rice culturability have mainly been performed on cultivated rice, and few studies have employed common wild rice (*Oryza rufipogon* Griff.). In this study, we isolate and characterize a gene responsible for mitigating callus browning in rice, named *BROWNING OF CALLUS1* (*BOC1*). The *BOC1* allele is specific to common wild rice. *BOC1* is an allele of a rice homolog of SRO (SIMILAR TO RCD ONE), termed *OsSRO1c*. The appropriate upregulation of *BOC1* significantly reduces callus browning during callus proliferation. *BOC1* is upregulated in common wild rice callus due to the presence of the *Tourist* MITE in the promoter region of *BOC1*. Additionally, *BOC1* improves the genetic transformation efficiency and frequency of *Hygromycin* (*Hyg*)-resistant calli following gene transfer. *BOC1* may regulate oxidative stress and programmed cell death (PCD), which may repress the callus browning. Our findings not only elucidate the mechanisms of callus browning during tissue culture, but also provide important insights into establishing a suitable genetic transformation system for the molecular breeding of rice.

## Results

### Phenotypic analysis of introgression line YIL25

**Fig. 1:**
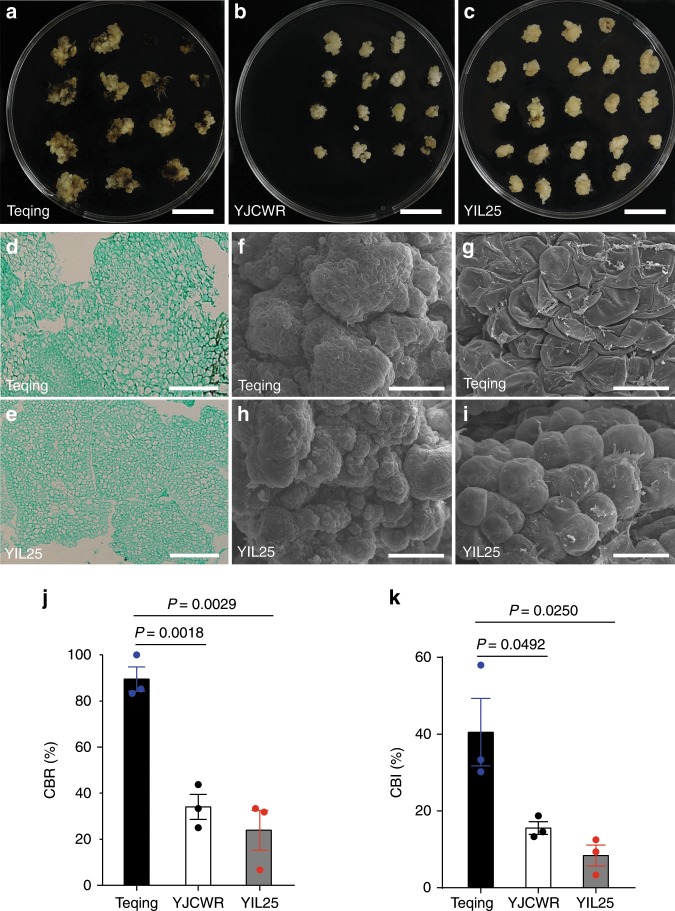
Phenotypic analysis of calli subcultured for 21 days. **a**–**c** Callus browning phenotypes observed in the calli after subculture for 21 days including **a** *indica* variety Teqing, **b** Yuanjiang wild rice (*Oryza rufipogon*, YJCWR), and **c** the introgression line YIL25. Scale bars, 2 cm. **d**, **e** Paraffin sections of **d** Teqing and **e** YIL25. Scale bars, 100 μm. **f**–**i** Scanning electron microscopy of Teqing and YIL25 calli. **f** Teqing calli. Thirty calli each from Teqing and YIL25 were collected for analysis of paraffin sections and scanning electron microscopy. Scale bars, 300 μm. **g** Enlarged from (**f**). Scale bars, 40 μm. **h** YIL25 calli. Scale bars, 300 μm. **i** Enlarged from (**h**). Scale bars, 40 μm. **j**, **k** Comparison of the CBR and CBI values of various calli. Values in (**k**, **l**) are means ± SE (*n* = 3 biologically independent samples). Two-tailed Student’s *t*-tests were performed to determine significant differences. Source data underlying Figs. **j**, **k** are provided as a Source Data file.

To identify the gene responsible for callus browning in rice, we constructed a set of introgression lines using a Yuanjiang common wild rice (YJCWR, *O. rufipogon*) accession that is relatively resistant to browning (Fig. 1b) as a donor and an elite *indica* cultivar Teqing (*O. sativa*) that is more susceptible to callus browning (Fig. 1a) as the recipient. We screened the introgression line YIL25 by inoculating mature seeds on unimproved NB (UINB) medium (NB basal medium without additives) for 7 days, and transferred the resulting scutellum-derived calli to UINB medium for 3 weeks of subculture (Fig. 1c). The callus browning rate (CBR) and callus browning index (CBI) were lower in YIL25 than Teqing (Fig. 1j, k). YIL25 contained three YJCWR chromosomal segments on the short arm of chromosomes 2, 3, and 5 (Supplementary Fig. 1). We, therefore, chose YIL25 as a reduced-browning line to cross with Teqing. The F_1_ plants showed similar phenotypes of CBR and CBI to YIL25 (Supplementary Fig. 2). These results indicated that the reduced-browning trait is controlled by a dominant gene. The histological examination indicated that the cell arrangement of YIL25 calli was more even and the cells were smaller and denser compared to Teqing (Fig. 1d, e). Observations using scanning electron microscopy (SEM) revealed that YIL25 calli consisted of many globular nodules (Fig. 1h) and the cells of YIL25 were turgid (Fig. 1i), whereas Teqing calli had fewer globular nodules (Fig. 1f) and the cells of Teqing were flaccid (Fig. 1g).

### Map-based cloning of the callus browning trait gene

**Fig. 2:**
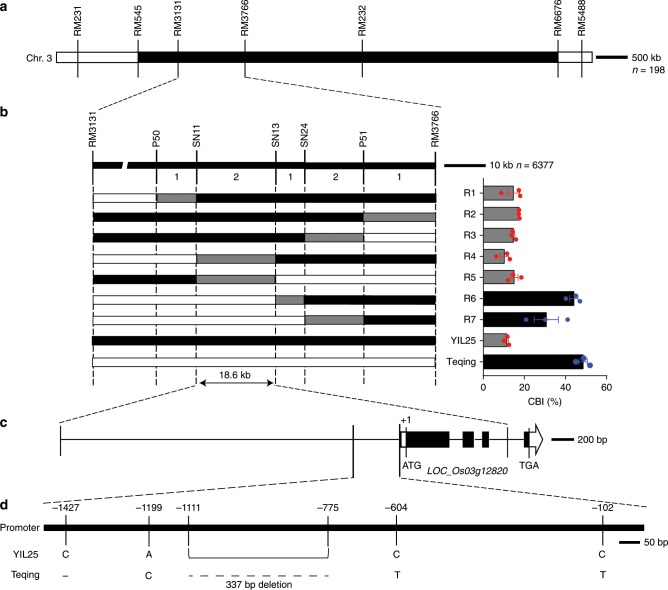
Map-based cloning of the callus browning trait gene. **a** *qCBT3* was initially localized to a region between markers RM3131 and RM3766 on the short arm of chromosome 3 (*n* = 198 plants of F_2_ populations derived from a cross between YIL25 and Teqing). Scale bar, 500 kb. **b** Positional cloning narrowed the *qCBT3* locus to an 18.6 kb region between markers SN11 and SN13 (*n* = 6377 individuals used for high-resolution mapping). R1 through R7 are recombinants. The black, white, and gray regions indicate homozygous for the YIL25 genome, homozygous for the Teqing genome, and the interval in the chromosome where crossover took place, respectively. Scale bar, 10 kb. *LOC_Os03g12820* is the candidate gene *BROWNING OF CALLUS1* (*BOC1*). Data are means (*n* = 3 biologically independent samples), with error bars showing standard error. **c** Structure of the candidate gene *BOC1*. White box, white box with arrow, black boxes, and lines between boxes indicate the 5′-UTR, 3′-UTR, exons, and introns, respectively. **d** Mutation sites of the *BOC1* promoter in YIL25 and Teqing. The ellipsis indicates deletion of bases. Scale bar, 50 bp. Source data underlying Figs. **a**, **b** are provided as a Source Data file.

To clone the gene for the callus browning trait, we constructed a F_2_ population of 198 segregating plants derived from a cross between YIL25 and Teqing. We used the CBI as a phenotypic index as it reflects the frequency and degree of callus browning. First, we measured the CBI on the seed-derived callus of the 198 individual plants. We then performed QTL analysis using simple sequence repeat (SSR) markers. The callus browning trait QTL, *qCBT3*, which is located between SSR markers RM3131 and RM3766 on the short arm of chromosome 3, had the largest effect, explaining 14% of the phenotypic variance associated with decreased CBI (Fig. 2a; Supplementary Table 1).

For high-resolution mapping, we selected F_2_ individuals that were heterozygous at the *qCBT3* locus (RM3131-RM3766), containing one chromosomal segment from Teqing and one from YJCWR by self-crossing to construct a segregating population to eliminate gene interference on introgression segments of chromosomes 2 and 5 (Fig. 2b). We identified plants with recombination events between markers RM3131 and RM3766 by genotyping the 6377 individuals in the population and evaluated the CBI of these recombinant individuals (Fig. 2b). We then developed single nucleotide polymorphism (SNP) markers and delimited *qCBT3* to an 18.6 kb region between SNP markers SN11 and SN13. This region contained only one predicted gene (*LOC_Os03g12820*) in the Nipponbare reference genome (The Rice Genome Annotation Project Database, Fig. 2c). Sequence analysis of the coding region in this interval revealed no sequence variation between Teqing and YIL25. Next, we compared the promoter sequences upstream of the translation start site between Teqing and YIL25 and identified one 337 bp deletion, one 1 bp deletion, and three SNPs in Teqing (Fig. 2d; Supplementary Fig. 3). Therefore, we chose *LOC_Os03g12820* as the candidate gene for *BROWNING OF CALLUS1* (*BOC1*).

### Functional analysis of *BOC1*

**Fig. 3:**
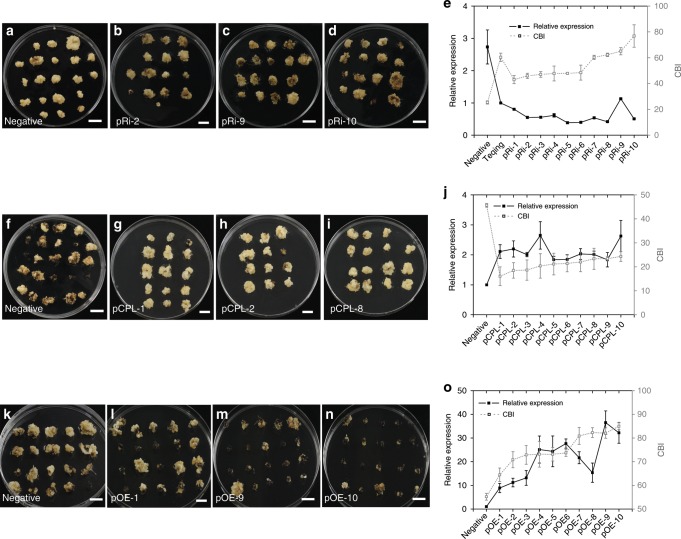
Functional analysis of *BOC1*. **a**–**d** Phenotypes of calli from pRi-*BOC1*-YIL25 transgenic plants (*n* = 18). Negative represents segregating plants from the transformation process but without the transgene. Scale bars, 1 cm. **e** Comparison of the relative expression levels and CBI between a negative transgenic line and 10 RNAi lines (*n* = 3 biological replicates). **f**–**i** Phenotypes of calli from pCPL-*BOC1*-Teqing transgenic plants (*n* = 15). Scale bars, 1 cm. **j** Comparison of the relative expression levels and CBI in a negative transgenic line and 10 complementation lines (*n* = 3 biological replicates). **k**–**n** Phenotypes of calli from pOE-*BOC1*-Teqing transgenic plants (*n* = 22). **o** Comparison of the relative expression levels and CBI in a negative transgenic line and 10 overexpression lines (*n* = 3 biological replicates). The black line indicates the trend of the relative expression levels, and the gray line indicates the trend of the CBI. Scale bars, 1 cm. Data are means ± SE, two-tailed Student’s *t*-tests. Source data underlying Figs. **e**, **j**, **o** are provided as a Source Data file.

To elucidate the function of *LOC_Os03g12820*, we constructed an RNA interference construct targeting *LOC_Os03g12820* and transformed it into introgression line YIL25 (designated as pRi-*BOC1*-YIL25). Eighteen transgenic lines were obtained. Reverse transcription-quantitative PCR (RT-qPCR) analysis showed that *LOC_Os03g12820* was expressed at lower levels in the pRi-*BOC1*-YIL25 positive transgenic lines than in the negative (non-transformed) plants (Fig. 3e). In addition, RNAi suppression of *LOC_Os03g12820* in the pRi-*BOC1*-YIL25 positive transgenic lines to the level of Teqing caused serious callus browning compared to the non-transformed controls (Fig. 3a–e).

Furthermore, we introduced a complementation vector containing the promoter, open reading frame and 3′-UTR of *LOC_Os03g12820* from YIL25 into Teqing (designated as pCPL-*BOC1*-Teqing) and obtained 15 positive transgenic plants. RT-qPCR analysis showed that the relative expression levels of *LOC_Os03g12820* were significantly elevated in the pCPL-*BOC1*-Teqing positive plants compared to the negative plants (Teqing) (Fig. 3j). The CBI was reduced in these positive lines when the expression level of *LOC_Os03g12820* was elevated at least 1.8 fold compared with the negative plants (Fig. 3f–j). This finding indicates that *LOC_Os03g12820* mitigates the callus browning phenotype. These results suggest that *LOC_Oso3g12820* is *BOC1* regulates callus browning in rice.

Next, we generated transgenic Teqing plants overexpressing the *LOC_Os03g12820* coding sequence of YIL25 (as shown in Supplementary Fig. 3, the rose red and red underline represent the nucleotide sequence of the overexpression construct, designated as pOE-*BOC1*-Teqing) and obtained 22 positive transgenic lines. RT-qPCR indicated that *BOC1* was strongly upregulated in the pOE-*BOC1*-Teqing positive transgenic lines compared to the negative plants (Fig. 3o). Nevertheless, when *BOC1* expression levels were 8.8-times higher in the tested pOE-*BOC1*-Teqing positive lines compared to the negative plants, callus browning was more severe (Fig. 3k–o). This result indicates that only the appropriate expression of *BOC1* reduces callus browning.

*BOC1* encodes an SRO protein of 463 amino acids, which includes a putative N-terminal RNA Recognition Motif (RRM) domain, a poly (ADP-ribose) polymerase (PARP)-like ADP-ribose transferase catalytic domain, and a C-terminal RST (RCD1, [RADICAL-INDUCED CELL DEATH1]—SRO [SIMILAR TO RCD ONE]—TAF4 [TBP-ASSOCIATED FACTOR4] (RST) domain (https://www.ncbi.nlm.nih.gov/Structure/cdd/wrpsb.cgi; Supplementary Fig. 4a). To evaluate the conservation of BOC1 in other plant species, we performed protein sequence alignment using the full-length protein sequence of BOC1 as a query in BLAST analysis and selected 24 putative homologs (sharing > 80% sequence identity with BOC1 at the nucleotide level) to construct a phylogenetic tree. Phylogenetic analysis indicated that BOC1 is closely related to other monocots, such as *Oryza brachyantha*, *Brachypodium distachyon*, *Triticum urartu*, and *Aegilops tauschii* (Supplementary Fig. 4b).

### Transcriptional characterization of *BOC1*

**Fig. 4:**
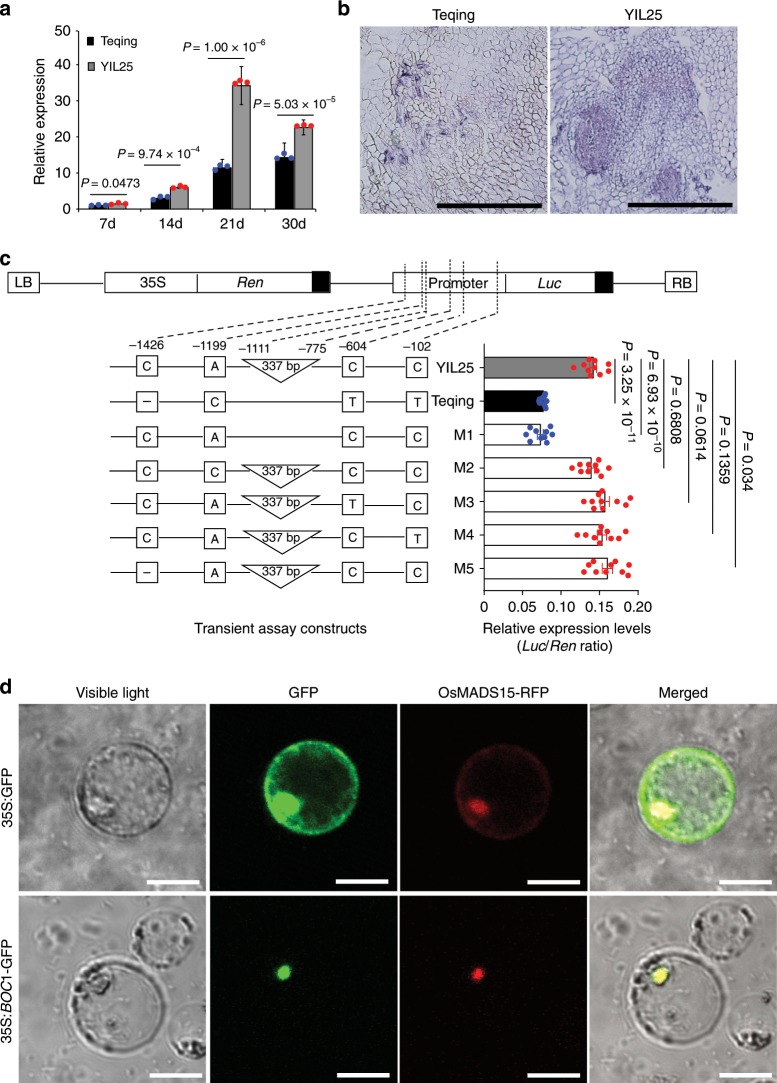
Transcriptional characterization of *BOC1*. **a** Expression analysis of *BOC1* in calli at different stages of subculture. Data are means, with bars showing SE, *n* = 3 biological replicates. Two-tailed Student’s *t*-tests were performed to determine significant differences. **b** *BOC1* expression patterns in Teqing and YIL25 calli subcultured for 21 days, as revealed by mRNA in situ hybridization. Thirty calli each from Teqing and YIL25 were collected for mRNA in situ hybridization. Scale bars, 100 μm. **c** Transient expression assays of the effects of InDels and SNPs in the *BOC1* promoter. Top, structure of the pGreenII 0800-*LUC* vector. Left, constructs with site-directed mutagenesis of the *BOC1* promoter. *Luc*, the target promoter controlling the *firefly luciferase* reporter gene. The *Renilla* (*Ren*) *luciferase* reporter gene was used as an internal control. M1 to M5 indicate the constructs with site-directed mutations at the 337 bp InDel, three SNPs, and 1 bp InDel. Right, expression levels of *Luc* relative to *Ren*. Data are presented as mean ± SE. Horizontal bars show the SE for all constructs (*n* = 10 biological replicates, two-tailed Student’s *t*-tests). **d** Subcellular localization of BOC1 protein in rice protoplast cells. OsMADS15 is a nuclear localization marker. Scale bars, 20 μm. *P*-values of (**a**, **c**) were calculated with two-tailed Student’s *t*-tests. The subcellular localization assays were performed with three biologically independent experiments. Source data underlying Figs. **a**, **c** are provided as a Source Data file.

To elucidate the expression profiles of *BOC1*, we analyzed the expression pattern of *BOC1* in different organs by RT-qPCR. The expression level of *BOC1* was highest in YIL25 calli after 21 days of subculture, followed by the tiller base, roots, culms, and leaves, and it was lowest in young panicles (Supplementary Fig. 5). *BOC1* expression increased gradually in Teqing and YIL25 throughout tissue culture. *BOC1* expression did not significantly differ between Teqing and YIL25 during the early stage of tissue culture, but significant differences were observed during the later stage; *BOC1* expression was notably higher in YIL25 than in Teqing in callus after 21 days of subculture (Fig. 4a). We further examined the expression of *BOC1* in calli after 21 days of subculture by RNA in situ hybridization and observed that the hybridization signals were stronger in YIL25 than in Teqing, confirming the notion that *BOC1* is expressed at higher levels in YIL25 callus (Fig. 4b).

Sequencing analysis revealed one 337 bp deletion, one 1 bp deletion, and three SNPs in the promoter region of *BOC1* between Teqing and YIL25 (Fig. 4c). To determine whether these deletions and SNPs in the *BOC1* promoter region directly contribute to its expression, we generated various constructs by inserting different combinations of mutant promoter fragments of YIL25 into binary vector pGreenII 0800-*LUC*. We introduced these constructs into rice protoplasts produced from callus that had been cultured for 21 days and examined the expression of the *luciferase* reporter gene (*LUC*). As shown in Fig. 4c, no significant differences in activity were observed between the YIL25 promoter fragment and mutated promoter fragments containing three SNPs (constructs M2–M4) and one 1 bp deletion (construct M5). However, the 337 bp deletion (construct M1) significantly decreased the promoter activity of *BOC1* to a level similar to that of Teqing (Fig. 4c). These results suggest that the 337 bp deletion is responsible for the reduced expression level of *BOC1* in Teqing calli. Using the RepeatMasker Web Server (http://www.repeatmasker.org/cgi-bin/WEBRepeatMasker), we examined the rice transposable element database using the 337 bp sequence as a query and determined that it is the *Tourist*-like miniature inverted-repeat transposable element (*Tourist* MITE), which belongs to the *PIF/Harbinger* superfamily. In addition, using PLACE (Plant *Cis-*acting Regulatory DNA Elements; http://www.dna.affrc.go.jp/htdocs/PLACE/), we identified three DOFCOREZM (AAAG, DOF TFBS)^25^, one MYB1AT (TAACCA, MYB TFBS)^26^, one MYCCONSENSUSAT (CAGATG, MYC TFBS)^26–28^, and one ACGTATERD1 (ACGT, ACGT TFBS)^28^ in this sequence; these elements are associated with plant stress responses (Supplementary Fig. 6). Subcellular localization analysis indicated that the BOC1-GFP fusion protein was specifically targeted to the nucleus in rice protoplasts (Fig. 4d).

### *BOC1* influences genetic transformation efficiency

**Fig. 5:**
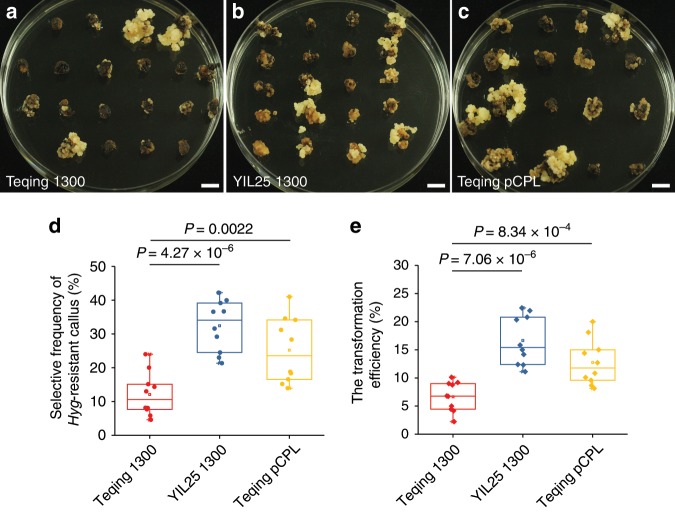
*BOC1* influences genetic transformation efficiency. **a**–**c** Transformed calli after three rounds of selection on *Hyg-*resistant UINB medium. Scale bars, 1 cm. **d** Comparison of the selection frequency of *Hyg*-resistant callus. Each dot represents the selective frequency of *Hyg*-resistant callus of 10 independent experiments. (**e**) Comparison of the transformation efficiency. Each diamond represents the transformation efficiency of 10 independent experiments. Teqing 1300, Teqing calli were infected with *Agrobacterium* harboring the binary pCAMBIA1300 empty vector as a control. YIL25 1300, YIL25 calli transformed with the binary vector pCAMBIA1300. Teqing pCPL, Teqing calli infected with *Agrobacterium* harboring the pCAMBIA1300-*BOC1* construct. The hollow box in each column in (**d**, **e**) represents the means of the selective frequency of *Hyg*-resistant callus and the transformation efficiency of 10 replicates, respectively. Box edges represent the 0.25 and 0.75 quantiles, with the median values shown by red, blue and yellow lines, respectively. Whiskers extend to data no more than 1.5 times the interquartile range, and the remaining data are indicated by dots. *P*-values of (**d**, **e**) were determined by two-tailed Student’s *t*-tests. Source data are provided as a Source Data file.

To determine if *BOC1* enhances the transformation efficiency of rice due to decreased callus browning, we transformed the binary vector pCAMBIA1300 into YIL25 (YIL25 1300), Teqing (Teqing 1300), and Teqing 1300 as a control. The pCAMBIA1300-*BOC1* complementation construct from YIL25 was transformed into Teqing (Teqing pCPL) by *Agrobacterium tumefaciens*-mediated transformation and cultured on UINB medium for *hygromycin* (*Hyg*) selection. The selection frequency of *Hyg*-resistant calli was more than 2.7-fold higher in YIL25 1300 than in Teqing 1300 (Fig. 5a, b, d), and the transformation efficiency was 2.5-fold higher in YIL25 1300 than in the Teqing 1300 control (Fig. 5e), indicating that YIL25 containing *BOC1* is an excellent receptor material for genetic transformation. Meanwhile, the selection frequency of *Hyg*-resistant calli was 2.1-fold better (Fig. 5a, c, d) and the transformation efficiency was 1.9-fold better in Teqing pCPL compared to the Teqing 1300 control (Fig. 5e). These results indicate that *BOC1* improves the selection frequency of *Hyg*-resistant calli and the genetic transformation efficiency of rice by reducing callus browning during *Agrobacterium*-mediated transformation.

### Physiological indices related to oxidative stress

**Fig. 6:**
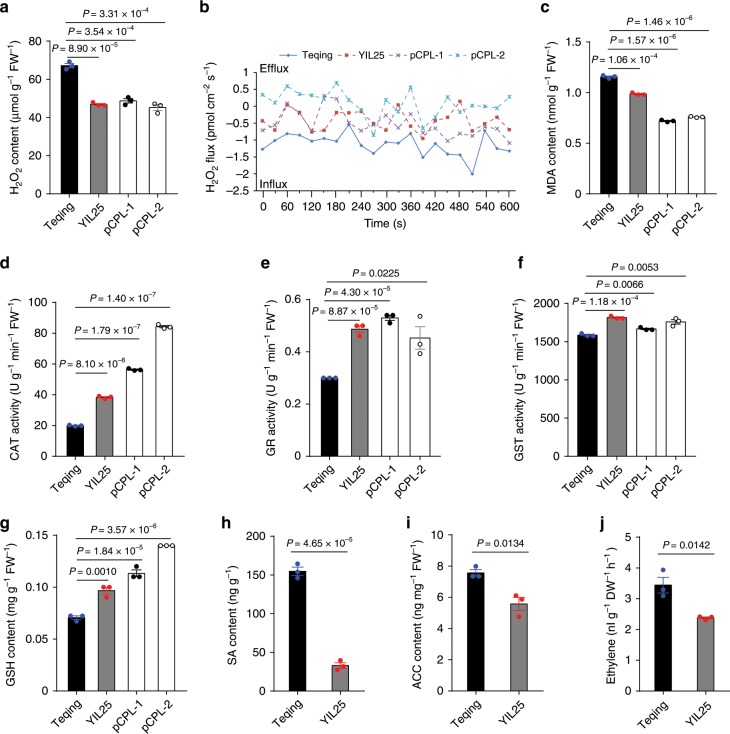
Physiological and biochemical oxidative stress indices of calli subcultured for 21 days. **a** H_2_O_2_ contents in the two parents and two positive complementation transgenic plants. **b** H_2_O_2_ flux (*n* = 4). **c** MDA contents. **d**–**f** Assay of antioxidant enzyme activities. CAT, catalase. GR, glutathione reductase. GST, glutathione S-transferase. **g** Antioxidant GSH contents. **h**–**j** Comparison of phytohormone contents between Teqing and YIL25. SA, salicylic acid; ET, ethylene; ACC, 1-aminocyclopropane-1-carboxylic acid, a direct precursor of ET. All values in (**a**) and (**c**) to (**j**) are presented as means ± SE (*n* = 3 biologically independent samples). *P*-values were analyzed by two-tailed Student’s *t*-tests. Source data are provided as a Source Data file.

To further explore the molecular mechanisms of callus browning, we measured various physiological and biochemical indices in calli at 21 days of subculture. Compared to Teqing, the H_2_O_2_ contents were lower in both YIL25 and pCPL calli and higher in pOE (pOE-*BOC1*-Teqing) calli (Fig. 6a; Supplementary Fig. 7a). In addition, we used Noninvasive Microtest Technology (NMT) on rice callus to detect intercellular H_2_O_2_ fluxes. There was a significant efflux of intracellular H_2_O_2_ in YIL25 and pCPL calli, whereas Teqing calli exhibited a significant influx of extracellular H_2_O_2_, suggesting that H_2_O_2_ was expelled from YIL25 and pCPL cells to alleviate celluar damage (Fig. 6b). MDA (malondialdehyde) is a biomarker of lipid peroxidation and an indicator of reactive oxygen species (ROS) production. Compared to Teqing, MDA contents were notably lower in the YIL25 and pCPL lines but higher contents in the pOE lines (Fig. 6c; Supplementary Fig. 7b), reflecting less damage from H_2_O_2_ in YIL25 and pCPL plants. Moreover, catalase (CAT), glutathione reductase (GR), and glutathione S-transferase (GST) activity and antioxidant glutathione (GSH) contents were higher in YIL25 and pCPL vs Teqing callus (Fig. 6d–g). However, GST activity and GSH contents were lower in pOE lines than in Teqing callus although CAT and GR activity did not significantly differ among lines (Supplementary Fig. 7c-f).

Finally, the levels of the plant hormones salicylic acid (SA) and ethylene (ET), as well as the direct ET precursor, 1-aminocyclopropane-1-carboxylic acid (ACC), were significantly lower in YIL25 compared to Teqing (Fig. 6h–j). Collectively, these findings suggest that BOC1 may inhibit PCD caused by oxidative stress, thereby reducing callus browning.

### RNA-seq analysis of *BOC1*

**Fig. 7:**
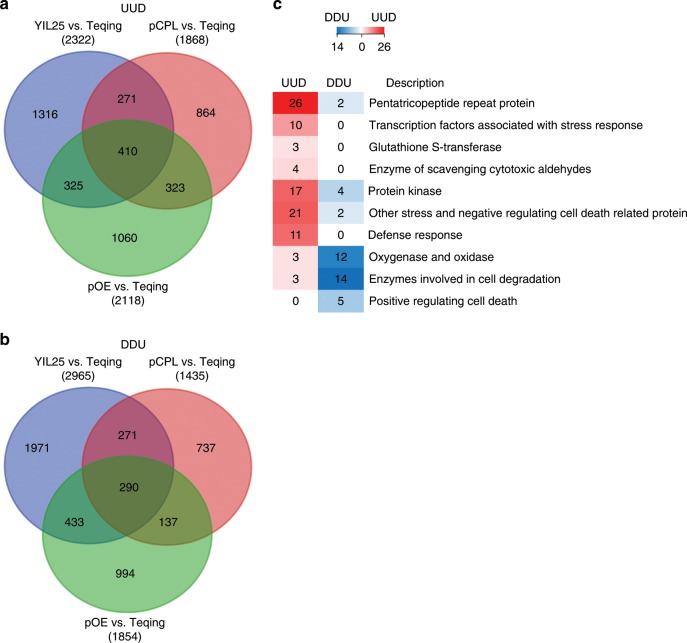
Differentially expressed genes in YIL25, the complementation line, overexpression line, and Teqing revealed by RNA-seq analysis. **a** Hierarchical clustering of 410 differentially expressed genes (DEGs) upregulated in YIL25 compared to Teqing, DEGs upregulated in the complementation line (pCPL) compared to Teqing, and DEGs downregulated in the overexpression line (pOE) compared to Teqing (UUD). **b** Hierarchical clustering of 290 DEGs downregulated in YIL25 compared to Teqing, DEGs downregulated in pCPL compared to Teqing, and DEGs upregulated in pOE compared to Teqing (DDU). **c** Hierarchical clustering of DEGs associated with stress response and cell death. The numbers in the boxes represent the number of genes per cluster.

To analyze the molecular function of *BOC1*, we performed RNA-seq experiments using callus that had been subcultured for 21 days from Teqing and YIL25, Teqing and the complementation line pCPL-*BOC1*-Teqing (pCPL), and also Teqing and the overexpression line pOE-*BOC1*-Teqing (pOE). We identified 700 differentially expressed genes (DEGs), including 410 DEGs that were upregulated in YIL25 versus Teqing callus and pCPL versus Teqing callus and downregulated in pOE versus Teqing callus (UUD, Fig. 7a, Supplementary Data 1). The 290 remaining DEGs were downregulated in YIL25 versus Teqing callus and pCPL versus Teqing callus and upregulated in pOE versus Teqing callus (DDU, fold change ≥ 2, FDR < 0.001) (Fig. 7b, Supplementary Data 2).

Among the UUD genes, 98 genes were involved in stress responses and cell death (Fig. 7c). Notably, these included 26 pentatricopeptide repeat protein genes, whose dysfunction leads to ROS accumulation;^29,30^ these genes were upregulated in YIL25 and pCPL but downregulated in pOE (Fig. 7c; Supplementary Fig. 8a). GST conjugates with GSH to scavenge ROS in order to protect cellular components from damage. In the current study, 3 GST genes present in the UUD DEG set and absent in the DDU DEG set (Fig. 7c; Supplementary Fig. 8c). In addition, compared to Teqing, GST activity and GSH contents were higher in YIL25 and pCPL callus but lower in pOE callus (Fig. 6f, g; Supplementary Fig. 7d,f). Among the UUD genes were four aldehyde dehydrogenase genes; aldehyde dehydrogenase enhances tolerance to various abiotic stresses by scavenging cytotoxic aldehydes (Supplementary Fig. 8d), and MDA contents were higher in of Teqing and pOE callus compared to YIL25 and pCPL callus (Fig. 6c; Supplementary Fig. 7b). *LOC_Os01g67980*, which negatively regulates the senescence process, encodes senescence-associated protein OsSAG12-1. RNAi transgenic lines declined the expression of *OsSAG12-1* and developed early senescence^31^ (Fig. 7c; Supplementary Fig. 8f). In addition, the UUD genes included transcription factor genes associated with stress and defense responses, which might function in maintaining ROS homeostasis (Fig. 7c; Supplementary Fig. 8b, g).

Twelve DDU genes were associated with oxygenase and oxidase; cytochrome *P*450 has monooxygenase activity as a source of ROS^32^ (Supplementary Fig. 8h). The DDU group also included genes encoding aspartic proteinases, peptidases, and nuclease (involved in cell degradation), as well as genes encoding polygalacturonase, endoglucanase, and glycosyl hydrolases (involved in cell wall degradation) (Fig. 7c; Supplementary Fig. 8i). Emerging evidence suggests that aspartic proteases are involved in plant development and cell death^33^. Finally, five DDU DEGs encode positive regulators of the senescence process (Fig. 7c). These DEGs include *LOC_Os01g53260*, encoding *WRKY* transcription factor OsWRKY23, which positively regulates the senescence process^34^, and *LOC_Os12g36850*, encoding a pathogenesis-related bet v I family protein similar to probenazole-inducible protein PBZ1, which induces cell death in rice, tobacco, and *Arabidopsis* via its RNase activity inside the cell^35^ (Supplementary Fig. 8j). When we treated callus that was subcultured for 7 days with 0.5% H_2_O_2_ for 18 h, *PBZ* (*LOC_Os12g36850*) was upregulated compared to the untreated control (Supplementary Fig. 8k).

Taken together, these findings suggest that the appropriate expression of *BOC1* may be beneficial for rice cells to be in reducing state, prevented various stress, and then inhibited cell death.

### *BOC1* promoter sequence variation affects callus browning

**Fig. 8:**
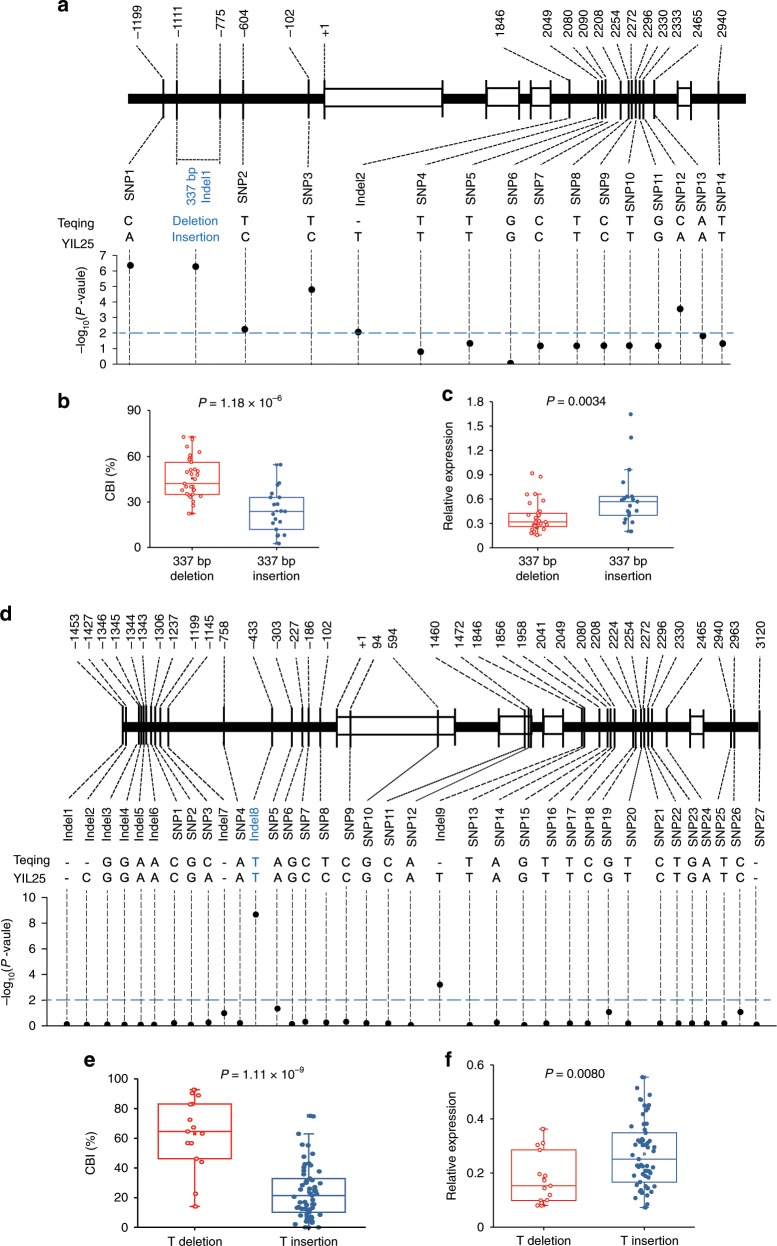
Association test and expression levels of loci with natural variations in *BOC1*. **a** Association testing of callus browning in 50 *O*. *rufipogon* accessions vs. 16 variant sites in the 4.6 kb genomic region. Black dots represent 16 variations. **b** Comparison of CBI in calli subcultured for 21 d in 31 accessions with the 337 bp deletion vs. 19 accessions with the 337 bp insertion. **c** Comparison of *BOC1* expression in calli subcultured for 21 d in lines harboring the 337 bp deletion vs. the 337 bp insertion (*n* = 3 biological replicates). **d** Association testing of callus browning in 74 Asian rice cultivars with 36 variant sites in the 4.6 kb genomic region. **e** Comparison of CBI in calli subcultured for 21 d between 59 accessions with the T deletion and 15 accessions with the T insertion. **f** Comparison of *BOC1* expression in callus subcultured for 21 d from rice varieties harboring the T deletion vs. the T insertion (*n* = 3 biological replicates). Box edges of (**b**, **c**) and (**e**, **f**) represent the 0.25 quantile and 0.75 quantile with the median values shown by red and blue lines. Whiskers extend to data no more than 1.5 times the interquartile range, and remaining data are indicated by dots. *P*-values of (**b**, **c**) and (**e**, **f**) were calculated by two-tailed Student’s *t*-tests. Source data underlying Figs. **b**, **c**, **e**, **f** are provided as a Source Data file.

To investigate the relationships between mutations in the *BOC1* locus and callus browning, we sequenced a ~4.6 kb genomic fragment covering the entire *BOC1* gene (2740 bp), the 1452 bp 5′-flanking region, and the 380 bp 3′-flanking region, from 50 *O. rufipogon* accessions (Supplementary Data 3) and 74 Asian rice cultivars (28 *japonica* and 46 *indica* cultivars, Supplementary Data 4). Nucleotide alignment showed that only 19 *O*. *rufipogon* accessions contained the *Tourist* MITE (Supplementary Data 3); however, the element at this locus was absent in all Asian rice cultivars examined (Supplementary Data 4). Therefore, we performed an association test of callus browning after 21 d of subculture vs. sequence variations in the *O. rufipogon* accessions and Asian rice cultivars. The association test of the common wild rice revealed the strongest association signals at the *Tourist* MITE and the four SNPs (Fig. 8a). Furthermore, we investigated the CBI of the 50 accessions of common wild rice and determined that this value was lower in the 19 accessions with the *Tourist* MITE than in the 31 accessions without this insertion (Fig. 8b). Moreover, the *BOC1* gene was expressed at higher levels in the 19 accessions with the *Tourist* MITE than in the 31 accessions without the *Tourist* MITE (Fig. 8c; Supplementary Data 3). In addition, we obtained previously reported the genome resequencing data for 446 *Oryza rufipogon* accessions^36^ and recovered the reads that mapped to the *Tourist* MITE flanking sequence in these accessions. We detected this site in 226 accessions (48 accessions with this MITE insertion and 178 accessions without this insertion). Forty-one of the 48 accessions with this insertion were in group III (closer to *japonica*) and 7 were in group II (the intermedia group between *indica* and *japonica*) (Supplementary Fig. 9a). By contrast, the 178 accessions without this MITE insertion were distributed among all the three groups including 65 in group I (closer to *indica*), 49 in group II, and 64 in group III (Supplementary Fig. 9a).

Nucleotide alignment of the 74 Asian rice cultivars revealed 9 Indels and 27 SNPs (Fig. 8d; Supplementary Data 4). To ascertain whether *BOC1* was responsible for callus browning in Asian rice cultivars, firstly, an association analysis was carried out between callus browning and sequence variations of 74 Asian rice cultivars. The result indicated that the Indel8 (−433, T indel) in the promoter of *BOC1* was the strongest signals (Fig. 8d). However, this site was no polymorphic in the 50 accessions of *O. rufipogon* (Supplementary Data 3, 4). We further found that the CBI in 15 varieties with T insertion in the promoter of *BOC1* was lower than that in 59 varieties without this insertion (Fig. 8e; Supplementary Data 4). We also analyzed the expression levels in these varieties and the result showed that the expression levels of *BOC1* was higher in the 15 varieties with T insertion in the promoter of *BOC1* than that in 59 varieties without this insertion (Fig. 8f; Supplementary Data 4). The above results suggest that the sequence variation in the *BOC1* promoter influence callus browning by affecting the expression level of *BOC1*.

Finally, we analyzed the fixation index (*F*_ST_, the level of population differentiation), on chromosome 3 in 1083 cultivated *indica* and *japonica* rice varieties^36^. The *F*_ST_ level in *BOC1* was 0.21, indicating that no significant differences in *BOC1* exist between the *indica* and *japonica* subspecies (Supplementary Fig. 9b). Therefore, we propose that the effect of *BOC1* on callus browning is not related to the differences between the *indica* and *japonica* subspecies.

## Discussion

Here, we demonstrated that the proper expression of *BOC1* decreases callus browning and increases the transformation efficiency of rice. *BOC1* encodes an SRO (SIMILAR TO RCD ONE) protein that is allelic to OsSRO1c, a regulator of oxidative stress responses^37^. RCD1 plays an important role in regulating cell death by controlling oxidative stress responses in *Arabidopsis* responses^38–43^. *OsSRO1c* is the major stress-responsive gene in the SRO family. Both *ossro1c*-*1* mutants and amiRNA (artificial microRNA)-*OsSRO1c* plants are hypersensitive to drought stress at the seedling stage. In addition, *OsSRO1c*-overexpressing rice plants showed more severe damage under drought stress than wild-type plants, whereas plants overexpressing *SNAC1*, encoding a NAC transcription factor that directly regulates *OsSRO1c* expression, showed increased drought resistance^29^. In the current study, RNA interference and *BOC1* overexpression plants showed a callus-browning phenotype similar to or more severe than that of Teqing, whereas lines harboring the complementation vector exhibited reduced callus browning, indicating that the appropriate upregulation *BOC1* reduces callus browning.

Transposable elements, among the most variable components of the genome, can replicate and integrate into positions, which affects the expression levels of adjacent genes, thereby resulting in genetic diversity^44^. For example, the terminal-repeat retrotransposon in miniature (TRIM) elements insertion in the promoter of *MS2* allele in the male-sterile *ms2* mutant resulted in an anther-specific expression pattern in wheat^45^. In maize, an 82 bp miniature inverted-repeat transposable element (MITE) into the promoter of *ZmNAC111* represses its expression; this MITE is significantly associated with natural variation^46^. In the current study, the presence of *Tourist* MITE in the *BOC1* promoter significantly increased *BOC1* expression in rice callus.

Callus cultured in vitro is subjected to complex growth conditions compared to cells in plants. Callus cells are exposed to the oxygen in air, which tends to result in oxidative stress. Oxidative browning is a prevalent problem in plant tissue culture that can cause cell death, resulting in brown tissues^47^. A moderate increase in the levels of the ROS H_2_O_2_ helps protect plant cells from subsequent exposure to more severe abiotic or oxidative stress^48^. However, the excessive accumulation of H_2_O_2_ might be involved in regulating PCD^38–40,49–51^. Moreover, high levels of H_2_O_2_ induce the accumulation of SA, resulting in severe oxidative damage^39,52^; in turn, SA pretreatment mediates the accumulation of H_2_O_2_^53^. Endogenous ET in in vitro culture may inhibit the synthesis of protective enzymes, such as CAT, for scavenging H_2_O_2_, which is involved in senescence, browning, and necrosis of callus^54^. In the current study, YIL25 callus contained decreased levels of H_2_O_2_ and the phytohormones ET and SA, as well as increased levels of CAT, GR, and GST activity compared to Teqing. The RNA-seq data analysis revealed that *BOC1* influences the expression of genes associated with ROS production and scavenging, stress response and cell death. In recently report, RCD1 coordinates chloroplast and mitochondrial functions through interaction with transcription factors. RCD1 participates in regulation of mitochondrial respiration and chloroplast ROS processing and may contribute to the survival of plant under a changing environment^55^. However, as shown in Supplementary Fig. 4b, RCD1 and OsSRO1c have different evolutionary histories which may have contrasting functions in the regulation of ROS related processes. In the current study, we use the calli subcultured for 21 d in the dark and is not necessary for photosynthesis by origin of carbon from medium. Therefore, the interaction and phenotype of BOC1 may be different from seedling stage, which will be an interesting research topic.

Common wild rice, the wild progenitor of cultivated rice, has an important gene pool that includes some alleles no longer present in cultivated rice^56^. The insertion of the 337 bp *Tourist* MITE in the *BOC1* promoter increases *BOC1* expression. This insertion is only detected in certain accessions of wild rice, but not in cultivated rice. Therefore, wild rice could be used to provide alleles for improving the tissue culture characteristics and genetic transformation efficiency of cultivated rice. Moreover, the genetic manipulation of the *BOC1* promoter in cultivated rice might also improve tissue culture ability. Perhaps the *BOC1* allele of wild rice and even similar alleles in other crops of the Gramineae family could be used to create recipient varieties more amenable to genetic transformation. The use of *BOC1* in in vitro rice callus propagation might help improve genetic transformation efficiency and selection to facilitate the molecular breeding of rice.

## Methods

### Plant materials

The wild rice introgression line YIL25, with reduced levels of callus browning, was derived from a cross between an *O*. *rufipogon* accession (YJCWR) as the donor and the elite *indica* variety Teqing (*O*. *sativa*) as the recipient^57^. The mapping population was developed from a cross between introgression line YIL25 and Teqing. The 50 common wild rice accessions and 74 *O*. *sativa* varieties (28 *japonica* and 46 *indica* varieties) used in this study are listed in Supplementary Data 3 and Supplementary Data 4.

### Tissue culture procedure

Mature, healthy, dehusked seeds were sterilized by immersion in 70% ethanol for ~2 min, followed by 15% sodium hypochlorite solution for 15 min with shaking, and rinsed three or four times with sterile water on an ultraclean workbench. Unimproved NB (UINB) medium (NB medium containing N6 macronutrient components^58^, B5 micronutrient components, and organic components^59^, supplemented with 2 mg/L of 2,4-D and 30 g/L of sucrose) was used for callus induction and subculture. The pH of the medium was adjusted to 5.8 with 1 N KOH, and 3 g/L of Phytagel was added before autoclaving at 121 °C for 20 min. Approximately 90 mature seeds per line, evenly distributed among three Petri dishes, were incubated in induction medium for 7 days at 28 °C in the dark. The induced calli were transferred to subculture (UINB) medium and incubated in the dark at 28 °C for 3 weeks. The traits of the calli in each Petri dish were recorded.

### Phenotype observations

The tendency for callus browning was categorized into four levels: (0) less than 1/10 of the callus tissue was brown (recorded as no browning); (1) 1/10 ~ 1/3 of the callus tissue was brown, considered light browning; (2) 1/3 ~ 2/3 of the callus tissue was brown, considered medium browning; (3) 2/3 ~ 1 of the callus tissue was brown, considered deep browning; and (4) the callus was completely brown. The tendency for callus browning, selection frequency of *Hyg-*resistant calli and transformation efficiency were calculated according to the following Eqs. (1, 2, 3, and 4):

1 $${\mathrm{Callus}}\;{\mathrm{browning}}\;{\mathrm{rate}}\;\left( {{\mathrm{CBR}}} \right) =\ 	\left( {{\mathrm{number}}\;{\mathrm{of}}\;{\mathrm{calli}}\;{\mathrm{showing}}\;{\mathrm{browning}}}\right./\\ 	\left.{{\mathrm{number}}\;{\mathrm{of}}\;{\mathrm{transferred}}\;{\mathrm{calli}}} \right) \times 100\%$$

2 $${\mathrm{Callus}}\;{\mathrm{browning}}\;{\mathrm{index}}\;\left( {{\mathrm{CBI}}} \right) =\ 	\left( {\Sigma \;{\mathrm{number}}\;{\mathrm{of}}\;{\mathrm{calli}}\;{\mathrm{at}}\;{\mathrm{each}}\;{\mathrm{browning}}\;{\mathrm{level}} \times {\mathrm{browning}}\;{\mathrm{level}}} \right)/ \\ 	\left ( {{\mathrm{number}}\;{\mathrm{of}}\;{\mathrm{transferred}}\;{\mathrm{calli}} \times {\mathrm{highest}}\;{\mathrm{browning}}\;{\mathrm{level}}} \right) \times 100\%$$

3 $${\mathrm{Selection}}\;{\mathrm{frequency}}\;{\mathrm{of}}\;Hyg \hbox{-} {\mathrm{resistant}}\;{\mathrm{calli}} =\ 	\left( {{\mathrm{number}}\;{\mathrm{of}}\;Hyg {\hbox{-}} {\mathrm{resistant}}\;{\mathrm{calli}}}\right./ \\ 	 \left. {{\mathrm{number}}\;{\mathrm{of}}\;{\mathrm{infected}}\;{\mathrm{calli}}} \right) \times 100\%$$

4 $${\mathrm{Transformation}}\;{\mathrm{efficiency}} =\ 	\left( {{\mathrm{number}}\;{\mathrm{of}}\;{\mathrm{independent}}\;{\mathrm{transgenic}}\;{\mathrm{events}}\;{\mathrm{regenerating}}\;{\mathrm{plants}}}\right./\\ 	 \left. {{\mathrm{number}}\;{\mathrm{of}}\;{\mathrm{transferred}}\;{\mathrm{calli}}} \right) \times 100\%$$

### Scanning electron microscopy

To prepare histological sections, calli that had been subcultured for 21 days were fixed in 2.5% glutaraldehyde-phosphate buffer saline fixative solution (pH 7.2) for more than 2 h and postfixed in the same buffer containing 1% OsO_4_. After being dehydrated through an ethanol series and dried using a carbon dioxide critical-point dryer, the calli were cleaned with ethanol and dried at 45 °C. The dry calli were gold plated and photographed under a Hitachi S3400 scanning electron microscope (Japan).

### Primers

The primers used in this study are listed in Supplementary Data 5.

### Genetic confirmation

The RNA interference construct was generated by inserting a hairpin sequence with a 350 bp and a 346 bp cDNA inverted repeat fragment of *BOC1* into the pTCK303 vector driven by the maize *Ubiquitin* promoter and transformed into YIL25 (pRi-*BOC1*-YIL25). A 4572 bp genomic fragment from YIL25 harboring the entire *BOC1* gene sequence with the 1452 bp 5′-flanking region and the 380 bp 3′-flanking region was amplified and inserted into binary vector pCAMBIA1300 (http://www.cambia.org) to form the complementary construct (pCPL). The overexpression vector (pOE) contained the 1392 bp YIL25-*BOC1*-ORF, which was amplified and inserted into the pCAMBIA1301 vector (http://www.cambia.org) driven by the *Ubiquitin* promoter. Both the pCPL and pOE constructs were transferred into Teqing and designated as pCPL-*BOC1*-Teqing and pOE-*BOC1*-Teqing, respectively. Relevant primer sequences are listed in Supplementary Data 5.

### RT-qPCR

Total RNA was extracted from various samples using TRIzol reagent (Life Technologies) and purified using an RNeasy Mini Kit (Qiagen) following the manufacturer’s instructions. First-strand cDNA was synthesized using Oligo(dT)_15_ primers (TaKaRa) and SuperScript III Reverse Transcriptase (Invitrogen) with 3 μg of total RNA. The qPCR was performed using a CFX96 Real-Time System (Bio-Rad), with each reaction containing 5 ng of first-strand cDNAs, 4 μM of gene-specific primers, and 5 μL of real-time PCR SYBR MIX (iQ™ SYBR® Green Supermix, Bio-Rad) under the following conditions: initial denaturation at 95 °C for 3 min followed by 40 cycles of 95 °C for 30 s, 58 °C for 30 s, and 72 °C for 30 s. The rice housekeeping gene *Actin* (*LOC_Os03g50885*) was used as an internal control to normalize the gene expression data using the relative quantification method (2^–ΔΔCT^)^60^. Each set of experiments was repeated three times. The primers used for RT-qPCR and cDNA amplification are listed in Supplementary Data 5.

### mRNA in situ hybridization

mRNA in situ hybridization was performed with the method of Javelle et al.^61^ with minor modifications. YIL25 and Teqing calli that had been subcultured for 21 days were fixed in 3.7% FAA solution at 4 °C overnight, dehydrated, and embedded in paraffin (Paraplast Plus, Fisher Scientific). The calli were sliced into 10-μm sections with a microtome (Leica RM2145). A 344 bp fragment of *BOC1* cDNA was amplified and used as the template to generate sense and antisense RNA probes, which were labeled using a DIG RNA labeling Kit (Roche) according to the manufacturer’s instructions. The hybridized slides were observed under a microscope (Leica DMR). Relevant primer sequences are listed in Supplementary Data 5.

### Luciferase assays

The promoter sequences upstream of the *BOC1* translation start site of YIL25 and Teqing were amplified using specific primers designed with site-directed mutations at the InDels and SNPs. The fragments were inserted into the pGreenII 0800-*LUC* vector to drive the expression of the *firefly luciferase* (*LUC*) gene. The effector plasmid DNA was transformed into rice protoplasts prepared from rice calli that had been subcultured for 21 days in the dark using the polyethylene glycol-mediated method^62^. The enzyme solution contained the following: 1.5% (w/v) “Onozuka” R-10 cellulose (Yakult Pharmaceutical Industry), 0.75% (w/v) macerozyme R-10 (Yakult Pharmaceutical Industry), 0.5 M D-mannitol, 2 mM 2-(N-morpholino) ethanesulfonic acid (MES; Sigma-Aldrich, cat. no. M3671), and 0.1 × W5 medium, pH 5.7. The protoplasts were lysed with Passive Lysis Buffer (PLB, Promega) and assayed using the Dual-Luciferase Reporter Assay System (Promega). Relevant primer sequences are listed in Supplementary Data 5.

### Subcellular localization

The entire coding sequence of *BOC1* was cloned into a *GFP* vector under the control of the *35* *S* promoter to generate *p35S*::*BOC1*-*GFP*. The RFP coding sequence was fused to the C terminus of *OsMADS15* to generate *OsMADS15*-*RFP* and was used as a nuclear marker. The two constructs were transformed into protoplasts and incubated at 28 °C in the dark for 16 h. Fluorescence was examined under a confocal laser scanning microscope.

### *Agrobacterium*-mediated genetic transformation

The binary plasmid vectors described above were transferred into *Agrobacterium tumefaciens* strain EHA105. Sterilized mature seeds were inoculated on UINB medium and cultured at 28 °C in the dark. After 7 days of incubating, the calli were collected and used for transformation. After 3 days of cocultivation with *Agrobacterium* at 25 °C in constant darkness on NB-Acetosyringone (AS) medium (30 g/L sucrose, 10 g/L glucose, 0.3 g/L casamino acids, NB medium salt mixture [Duchefa Biochemie], 0.5 mg/L glutamine, and 2.5 mg/L 2,4-D, 20 mg/L AS, pH 5.3), the calli were washed two or three times with sterile water and rinsed once with sterile water containing 200 mg/L Timentin (Caisson Labs, USA) and 200 mg/L cephalosporin (Wako Pure Chemical Industries). The calli were cultured on UINB medium containing 50 mg/L hygromycin B (Hyg, Merck), 200 mg/L Timentin, and 200 mg/L cephalosporin for three rounds of 15 days each at 28 °C in the dark. For regeneration, vigorously growing calli were transferred to MS regeneration medium (30 g/L sucrose, 30 g/L sorbitol, 2 g/L casamino acids, MS Medium Salt Mixture [Duchefa Biochemie], 1.1 g/L MES, 2 mg/L 1-naphthalene acetic acid, and 1 mg/L kinetin, pH 5.8) and cultured for 15 to 30 days at 28 °C (16 h light/8 h dark). The means in the genetic transformation efficiency test represent data from 10 groups of independent experiments, with ~80 to ~120 transformed calli per group.

### Quantitative determination of H_2_O_2_ contents and H_2_O_2_ flux

H_2_O_2_ concentrations were measured in calli subcultured for 21 days^63^. The calli (100 mg) were homogenized in 1 ml 0.1% (w/v) TCA in an ice bath. The homogenate was centrifuged at 12,000 × *g* for 15 min, and 1 ml of the supernatant was added to 1 ml 100 mM potassium phosphate buffer (pH 7.0) and 2 ml 1 M KI. The absorbance of the supernatant was read at 390 nm based on the fresh weight of the callus. H_2_O_2_ flux was measured using the Non-invasive Micro-test Technology (NMT) System (NMT100-SIM-XY, Younger USA Sci. & Tech. Corp., Amherst, MA, USA) at the Xuyue Beijing NMT Service Center (Xuyue Beijing Sci. and Tech. Co., Ltd., Beijing, China). Callus that had been subcultured for 21 d was fixed onto the bottom of a Petri using a filter paper strip and resin block. The callus in the Petri dish was incubated in measuring solution (0.1 mM KCl, 0.1 mM CaCl_2_, 0.1 mM MgCl_2_, 0.5 mM NaCl, 0.3 mM MES, 0.2 mM Na_2_SO4, pH 6.5) for ~20 min. The measuring solution was discarded and replaced with 5~10 ml of fresh measuring solution. H_2_O_2_ flux microsensor was placed a ~10 μm from the calli, and H_2_O_2_ flux at each site was measured for 10 min; each measurement repeated four times. Flux data were obtained using imFluxes V2.0 software (Younger USA LLC, Amherst, MA 01002, USA): a positive value represents efflux and a negative value represents influx. The H_2_O_2_ flux was calculated based on Fick′s law of diffusion (5):

5 $$J\left( {{\mathrm{pmol}}/{\mathrm{cm}}^2/s} \right) = - D_o \times \left( {dc/dx} \right)$$

where *J* is the analyte H_2_O_2_ flux (unit: pmol·cm^−2^·s^−1^), *dc* is its concentration gradient, *dx* is the distance between the two points (30 μm), and *D*_*0*_ is its diffusion constant (unit: cm^−2^·s^−1^).

### Measurement of lipid peroxidation

MDA was measured using the thiobarbituric acid (TBA) assay^64^. The calli (100 mg) were homogenized in 1 ml 0.1% (w/v) TCA in an ice bath. After centrifuging the homogenate at 12,000 × *g* for 10 min, 0.5 ml of the supernatant was combined with 0.5 ml 0.6% thiobarbituric acid (TBA) and incubated at 95 °C for 15 min. The samples were cooled immediately in an ice bath and the absorbance of the supernatant measured at 450, 532, and 600 nm.

### Assay of GSH content

GSH content was assayed by Shanghai Sanshu Biotechnology Co., Ltd (Shanghai, China). The reaction mixture contained plant extract, 0.1 M NaH_2_PO_4_ (pH 7.7), and DTNB (5,5-dithiobis-2-nitrobenzoic acid). Absorbance was recorded at 412 nm^65^.

### Enzyme activity assays

The enzyme activity assays were carried out by Shanghai Sanshu Biotechnology Co., Ltd (Shanghai, China). Catalase (CAT) activity was measured based on the consumption of H_2_O_2_ and the decrease in absorbance at 240 nm^66^. Glutathione reductase (GR) was assayed based on the decrease in *A*_340_ due to nicotinamide adenine dinucleotide phosphate (NADPH) oxidation. The reaction mixture of GR contained 1.0 ml of 0.1 M potassium phosphate buffer (pH 8.0) including 0.5 mM EDTA, 0.5 mM MgCl_2_,1 mM NADPH, 10 mM oxidized glutathione (GSSG), and 0.1 ml enzyme extract^67^. The glutathione S-transferase (GST) was assayed using the substrate 1-chloro 2,4-dinitrobenzene (CDNB), and the activity was detected spectrophotometrically based on the change in absorbance at 340 nm^68^.

### Endogenous hormone level measurements

The calli were ground into a powder in liquid nitrogen in a mortar and each sample (0.05 g) were transferred to a 1.5 ml screw-cap tube. After adding 50 μl of internal standards and 500 μl of extraction solvent (2-propanol: H_2_O: HCl = 2:1: 0.002) to each tube, the sample was incubated on a shaker at 900 × *g* for 30 min at 4 °C. After adding 1 ml trichloromethane, the sample was shaken for 30 min and centrifuged at 14,000 × *g* for 5 min at 4 °C to form two phases. A 1.2 ml sample of solvent from the lower phase was dried in a nitrogen evaporator, dissolved in 0.1 ml methanol and endogenous SA levels quantified by high-performance liquid chromatography-mass spectrometry (HPLC-MS/MS)^69^.

Ethylene was analyzed according to a published method^70^ with minor modifications. The calli were placed into 100-mL vials that were promptly sealed with a rubber plug and incubated at 28 °C for 48 h in the dark. Ethylene content in 2 ml of the headspace gas was analyzed with a gas chromatograph (GC-17A, Shimadzu, Japan).

### RNA-seq analysis

Total RNA was isolated from Teqing and YIL25, pCPL and Teqing, and pOE and Teqing calli (subcultured for 21 days) with three biological replicates. Paired-end libraries were constructed and sequenced on the Illumina HiSeq 2500 platform at the Novogene Company (China). The RNA-seq reads were mapped to the reference genome (Os-Nipponbare-Refrence-IRGSP-1.0, MSU7) using TopHat2 with default parameters, and the FPKM (fragments per kilobase of exon per million mapped reads) of each gene and the DEGs (fold change ≥ 2, FDR < 0.001) between YIL25 and Teqing, pCPL and Teqing, and pOE and Teqing were calculated^71^.

### Sequencing and data analysis

The ~4.6 kb genomic fragment covering the entire *BOC1* gene (2740 bp), the 1,452 bp 5′-flanking region, and the 380 bp 3′-flanking region were amplified using five PCR primer pairs and sequenced using the Sanger sequencing approach. A previously published dataset in 1,083 diverse rice accessions was used to calculate the fixation index (*F*_ST_) across chromosome 3 between *indica* and *japonica* varieties using VCFtools 0.1.15, with a 100-Kb window size^36,72^.

### Data analysis

A two-tailed Student’s *t*-test was performed using SPSS version 16 (SPSS Inc., Chicago, IL, USA). QTL analysis was performed using MapManager QTXb 20^73^. Amino acid sequences homologous to BOC1 were downloaded from the National Center for Biotechnology Information website (http://blast.ncbi.nlm.nih.gov/). Phylogenetic trees were constructed using the neighbor-joining algorithm in MEGA version 7 with 1000 bootstrap replications^74^. The association tests were analyzed by R v3.6.1.

### Reporting summary

Further information on research design is available in the Nature Research Reporting Summary linked to this article.

## Supplementary information

Supplementary Information

Peer Review

Reporting Summary

Description of Additional Supplementary Files

Supplementary Data 1-5

## Data Availability

Data supporting the findings of this work are available within the paper and its Supplementary Information files. A reporting summary for this Article is available as a Supplementary Information file. The datasets generated and analyzed during the current study are available from the corresponding author upon request. RNA-seq data for callus that had been subcultured for 21 days from Teqing and YIL25, Teqing and the complementation line pCPL-*BOC1*-Teqing, and Teqing and the overexpression line pOE-*BOC1*-Teqing was deposited in the National Center for Biotechnology Information (NCBI) under GEO accession number GSE142094. The source data underlying Figs. 1j, 1k, 2a, 2b, 3e, 3j, 3o, 4a, 4c, 5, 6, 8b, 8c, 8e, and 8f, as well as Supplementary Figs. 2, 5, 7, 8k, and 9 are provided as a Source Data file.

## Source data

Source Data
