# Supplementary material for: A common wild rice-derived BOC1 allele reduces callus browning in indica rice transformation
Source: Nat Commun. 2020 Jan 23;11:443. doi: 10.1038/s41467-019-14265-0 (PMC6978460; doi:10.1038/s41467-019-14265-0)
Supplement: Supplementary file 1 — Supplementary Information [file 41467_2019_14265_MOESM1_ESM.pdf]

A common wild rice-derived *BOC1* allele reduces callus browning in *indica* rice transformation

Zhang *et al*.

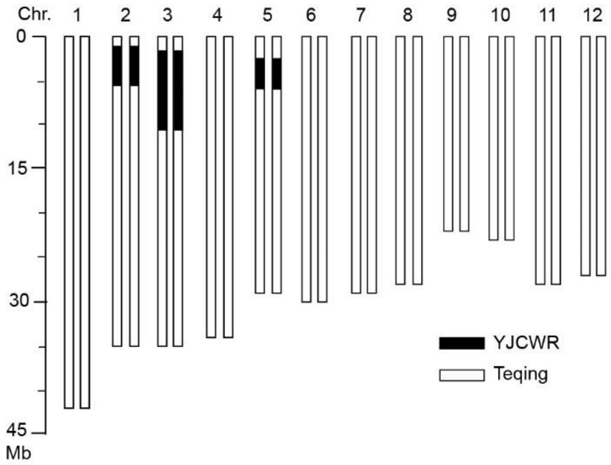

Supplementary Fig. 1. Graphic depiction of genotype of the introgression line YIL25. The black boxes represent the chromosome segments of YJCWR. The white boxes represent the Teqing genetic background.

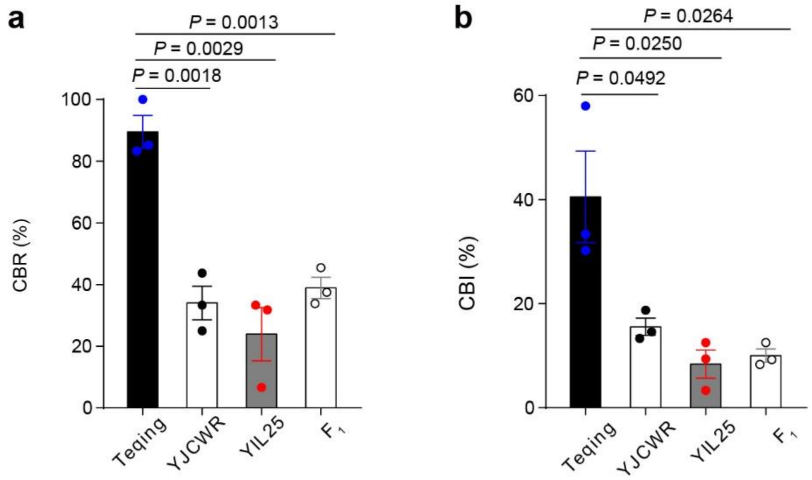

Supplementary Fig. 2. Phenotypic comparison of various calli subcultured for 21 days. (a, b) Comparison of the CBR and CBI of Teqing, YJCWR, YIL25, and F_1 progeny derived from a cross between Teqing and YIL25. Values are means ± SE (n = 3 biologically independent samples). Two-tailed Student's t-tests were performed to determine significant differences. Source data are provided as a Source Data file.

```
Teqing  AAAACTCCCGTAACCGTAGGATGATC-AGATGGTCAACGGCGATGGCAAAGGTCAGCGTTTGGTTTACCACGCCTTCGTC  79
YIL25   AAAACTCCCGTAACCGTAGGATGATCCAGATGGTCAACGGCGATGGCAAAGGTCAGCGTTTGGTTTACCACGCCTTCGTC  80

Teqing  CGGCGGTGGGCCGGTGGGGCCCACCTGGAAGTCTAATTATTACTAGACCGAGAAATAAACAACTTGCTTCACAAGAACAT  159
YIL25   CGGCGGTGGGCCGGTGGGGCCCACCTGGAAGTCTAATTATTACTAGACCGAGAAATAAACAACTTGCTTCACAAGAACAT  160

Teqing  CGTGGGTGGGAACGAGCGAAAGCAATCTTAAGCGCCAAGAAAAATACGCACAAGAGAGAAGAGTAGGCGCGGTTTTGTGG  239
YIL25   CGTGGGTGGGAACGAGCGAAAGCAATCTTAAGCGCCAAGAAAAATACGCACAAGAGAGAAGAGTAGGCGCGGTTTTGTGG  240

Teqing  CCGGAGAGAAAGCCACGCGCCGGTTTCGTGCCGGAGTATCATTTTCGTTTTCTTTTTTTCTCCCTTTCTTTTTTTCGCAT  319
YIL25   CCGGAGAGAAAGCAACGCGCCGGTTTCGTGCCGGAGTATCATTTTCGTTTTCTTTTTTTCTCCCTTTCTTTTTTTCGCAT  320

Teqing  TAACGGACGTTTCGTACTAA------------------------------------------------------------  340
YIL25   TAACGGACGTTTCGTACTAAGGCCCTGTTTAGATGGGACTAAAACTTTTAAGTCCCTATCATATCAGATGTTTGAAAATT  400

Teqing  --------------------------------------------------------------------------------  340
YIL25   AATTATAAATATTAAACGTAGACTATTAATAAAACCCATCCATAATCTTGGACTAATTTGCGAGACGAATCTAATGAGCC  480

Teqing  --------------------------------------------------------------------------------  340
YIL25   TAATTAATCCATGATTAGCCTATGTGATGCTACAGTAAACATTCTCTAATTATAGATTAATTAGGCTTAAAAAATTTGTC  560

Teqing  --------------------------------------------------------------------------------  340
YIL25   TCGTGAATTAGCTTTTATTTATGTAATTAGTTTTGTAAGTAGTCTATATTTAATACTCTAAATTAGTGTCTAAAGACAGA  640

Teqing  -------------------------------------GCTGGTATACAACAAGGAGACCGGCTGGGCGCCTGGGCCTGTC  382
YIL25   GACTAAAGTTAAGTCCCTGGATCTAATAACCACCTAAGCTGGTATACAACAAGGAGACCGGCTGGGCGCCTGGGCCTGTC  720

Teqing  CAGGTGAAGTAAAGGGCAAAGTCGGTACTAATCGGTATAGTCAGCGTGCTTCACATGCGACAACTCTGACCCAAGGTGAA  462
YIL25   CAGGTGAAGTAAAGGGCAAAGTCGGTACTAATCGGTATAGTCAGCGTGCTTCACATGCGACAACTCTGACCCAAGGTGAA  800

Teqing  GTAAAGAGCCAAGTCTTTATGTACGACAGTACGAGCAAGGACTAGTACTAGTAGAAGTGCTATGCTGAATTTTGCTATTG  542
YIL25   GTAAAGAGCCAAGTCTTTATGTACGACAGTACGAGCAAGGACTAGTACCAGTAGAAGTGCTATGCTGAATTTTGCTATTG  880

Teqing  TTAGGAAACAATTTTTTGGTTTATATAATTAACCAAACTCATACAATCAAATGTGTATGCATGAAATTGAAATGTAACCG  622
YIL25   TTAGGAAACAATTTTTTGGTTTATATAATTAACCAAACTCATACAATCAAATGTGTATGCATGAAATTGAAATGTAACCG  960

Teqing  TTTTTTAGACCAATTTTCTTATATTAATTAATTTATTATAGAGGAGAAATATTTTTTTCTTTTTTTAAGGGAAGGAGCAC  702
YIL25   TTTTTTAGACCAATTTTCTTATATTAATTAATTTATTATAGAGGAGAAATATTTTTTTCTTTTTTTAAGGGAAGGAGCAC  1040

Teqing  TATTCTTTCCGTTCAAGTAAGTAGTAGTCCAGACCAAAAAAGAAATAAAAAAACAACTGGCGCAAGTAAAACAGGTTCGG  782
YIL25   TATTCTTTCCGTTCAAGTAAGTAGTAGTCCAGACCAAAAAAGAAATAAAAAAACAACTGGCGCAAGTAAAACAGGTTCGG  1120

Teqing  GGGCAAACTAGACATTTCTAGACTTCTCTACCAAGGCATCTCCTTCTGTCCTTCATCAGGGGCAAAACCGCCATTACCAT  862
YIL25   GGGCAAACTAGACATTTCTAGACTTCTCTACCAAGGCATCTCCTTCTGTCCTTCATCAGGGGCAAAACCGCCATTACCAT  1200

Teqing  TACCAACAAACGCAAAGAAGACTCGGGCGGGCGCCGCCCCAGAACACAAGTGAGAAAAAAAAAAAAACCGACCTCCTCGCC  942
YIL25   TACCAACAAACGCAAAGAAGACTCGGGCGGGCGCCGCCCCAGAACACAAGTGAGAAAAAAAAAAAAACCGACCTCCTCGCC  1280

Teqing  GACGTGCTCTTCAAGAGACCGACCCCCCGCCGCGGCCTCAACCTATATATTCATCCCCACCTCCTCCGTCTTCCGCTCCC  1022
YIL25   GACGTGCTCTTCAAGAGACCGACCCCCCGCCGCGGCCTCAACCTATATATTCATCCCCACCTCCTCCGTCCTCCGCTCCC  1360

Teqing  ACTTGATCCCGATCGCCATTTCTCCACCTGCACATCTGCGCGCGCGCGCGGGAGAGCAGAGGCGGCGAGAGAGATCCGGG  1102
YIL25   ACTTGATCCCGATCGCCATTTCTCCACCTGCACATCTGCGCGCGCGCGCGGGAGAGCAGAGGCGGCGAGAGAGATCCGGG  1440

                   +1Translation initiation site
Teqing  TCGGGAGGGGTGATGGACTTCTCCGGCGACGTCAAGCCGGCGATCCACCGGCCCTCTGTCGCGGCGGCACGCGGGGGAGG  1182
YIL25   TCGGGAGGGGTGATGGACTTCTCCGGCGACGTCAAGCCGGCGATCCACCGGCCCTCTGTCGCGGCGGCACGCGGGGGAGG  1520

Teqing  GAACGGTGGGGCGATCCCGCTCCTGCGTGGGTGGCAGGCGTTCCGGAGGAGCGGCGCGCCGGCGAGGCTCCTCTGCTTCG  1262
YIL25   GAACGGTGGGGCGATCCCGCTCCTGCGTGGGTGGCAGGCGTTCCGGAGGAGCGGCGCGCCGGCGAGGCTCCTCTGCTTCG  1600

Teqing  AGGGCGGCGCGTGGGCGGACGTCGCGGGCGAGGTGGTGGGGCTGCTGCGGCGGGCGTTCATGGAGGGGAAGGCCGTTTGC  1342
YIL25   AGGGCGGCGCGTGGGCGGACGTCGCGGGCGAGGTGGTGGGGCTGCTGCGGCGGGCGTTCATGGAGGGGAAGGCCGTTTGC  1680

Teqing  GAGGCCGCCTGCGGTGGGAGGGTGTTCCTGTTCGACTTCATGCGGATGGTTCGGATCGATGAGGCCACCGCCGAGGAGGC  1422
YIL25   GAGGCCGCCTGCGGTGGGAGGGTGTTCCTGTTCGACTTCATGCGGATGGTTCGGATCGATGAGGCCACCGCCGAGGAGGC  1760

Teqing  CGCGCTGGGGTGGATCGACGACCGCGGCGCGTGCTTCTTCCCGGCTCCCGAGGGCGGGAGGAAGAGGAAGAGGGAGAGGG  1502
YIL25   CGCGCTGGGGTGGATCGACGACCGCGGCGCGTGCTTCTTCCCGGCTCCCGAGGGCGGGAGGAAGAGGAAGAGGGAGAGGG  1840

Teqing  ACGAGGCGGGGTCGGAGGTGAAGGGGGAGGATCGGCGGCGGCGGCAGCCGGCGGCGGAGGAGGAGGACGGGGACGAGGCG  1582
YIL25   ACGAGGCGGGGTCGGAGGTGAAGGGGGAGGATCGGCGGCGGCGGCAGCCGGCGGCGGAGGAGGAGGACGGGGACGAGGCG  1920
```

```
Teqing  TCGTCCGGCGTGGAGGAGCGGTCCGGGGAGAGCCGCCCCGAGGCGGATGAGCCCGACAGGAAGAAGGCGCGCGGGACGTT  1662
YIL25   TCGTCCGGCGTGGAGGAGCGGTCCGGGGAGAGCCGCCCCGAGGCGGATGAGCCCGACAGGAAGAAGGCGCGCGGGACGTT  2000

Teqing  GTGGGGGAAGGCGGTGAGGCTGGACGAGGCGGACAAGTTCTACAAGGTGGTGGAGAAGCTCTTCGTCAGCCGGATGGCTC  1742
YIL25   GTGGGGGAAGGCGGTGAGGCTGGACGAGGCGGACAAGTTCTACAAGGTGGTGGAGAAGCTCTTCGTCAGCCGGATGGCTC  2080

Teqing  CCGTGGCGGCGGCCCGCGGCGTGGCGATCACGGCGGTGCACAAGGTCGCGCAGGGGCCCCGGGCAAGAGCCTTCCATCTG  1822
YIL25   CCGTGGCGGCGGCCCGCGGCGTGGCGATCACGGCGGTGCACAAGGTCGCGCAGGGGCCCCGGGCAAGAGCCTTCCATCTG  2160

Teqing  CAGGGACAGCTCCTTGCCGCTGCTCGCGGCGTCGGCGATGGCAGCAACGCCAAGTTCGCGTGGTACGGCGCGCCGGCGGC  1902
YIL25   CAGGGACAGCTCCTTGCCGCTGCTCGCGGCGTCGGCGATGGCAGCAACGCCAAGTTCGCGTGGTACGGCGCGCCGGCGGC  2240

Teqing  GGATGTGGCCGCGGCGGTGGAGCACGGCTTCGGGAGGACGAACGGGCAGTTTCTCGGCGGGCGCGCACACGGCGACGGCG  1982
YIL25   GGATGTGGCCGCGGCGGTGGAGCACGGCTTCGGGAGGACGAACGGGCAGTTTCTCGGCGGGCGCGCACACGGCGACGGCG  2320

Teqing  TTCACCTTTCGCCGCCGCAGTACCCTCACGCTAGGTGAGTTTCCCAAATGCTTTCAACTCTTTCCATCTGCAAATGCTTT  2062
YIL25   TTCACCTTTCGCCGCCGCAGTACCCTCACGCTAGGTGAGTTTCCCAAATGCTTTCAACTCTTTCCATCTGCAAATGCTTT  2400

Teqing  GAACTATTGGGGAGAACATTTTTAGTAATTTCTGAAAATTCACCAAGTGCCTCATATATTTCATCTTTCGATATTACCAT  2142
YIL25   GAACTATTGGGGAGAACATTTTTAGTAATTTCTGAAAATTCACCAAGTGCCTCATATATTTCATCTTTCGATATTACCAT  2480

Teqing  ACGAATCTTGAGTAAAATTATTTTCTGCCTGCCATCTTGGTGCTAAACAATGAAACTACCAATCGTTGTTTTGCATCTAA  2222
YIL25   ACGAATCTTGAGTAAAATTATTTTCTGCCTGCCATCTTGGTGCTAAACAATGAAACTACCAATCGTTGTTTTGCATCTAA  2560

Teqing  GTTTGAATTTAGAGCAAATTAGACAGTATGGTACAGTAGCTACCTAAATTGTTATAAATTGGGATGAAATTTGTGAGTCA  2302
YIL25   GTTTGAATTTAGAGCAAATTAGACAGTATGGTACAGTAGCTACCTAAATTGTTATAAATTGGGATGAAATTTGTGAGTCA  2640

Teqing  ATGTTCAACTAAGTTAACGAGTTAATTAACTTGTCTTCGAGCAGTGCGATGCTGACCAAGCCAGACGAGAATGGCGAGGC  2382
YIL25   ATGTTCAACTAAGTTAACGAGTTAATTAACTTGTCTTCGAGCAGTGCGATGCTGACCAAGCCAGACGAGAATGGCGAGGC  2720

Teqing  ACACATCGTGCTGTGCCGCGTCCTGATGGGCCGTCCAGAGGCCGTCCCTGCCAGCTCACCCCAATTCCACCCCAGCAGCG  2462
YIL25   ACACATCGTGCTGTGCCGCGTCCTGATGGGCCGTCCAGAGGCCGTCCCTGCCAGCTCACCCCAATTCCACCCCAGCAGCG  2800

Teqing  ACGAATACGACAGCGCCGTCGACAACCTTGAGAATCCGCGGTGGTACGTTGTATGGAGCACAGACATGAACACCAGGATC  2542
YIL25   ACGAATACGACAGCGCCGTCGACAACCTTGAGAATCCGCGGTGGTACGTTGTATGGAGCACAGACATGAACACCAGGATC  2880

Teqing  CTCCCAGAGTACGTGGTCAGCTTCAGGTGGCCCAACCTGCCGCAGATGGAAGGTTGGTCTCTCGTGTCTGAAATTTGCTG  2622
YIL25   CTCCCAGAGTACGTGGTCAGCTTCAGGTGGCCCAACCTGCCGCAGATGGAAGGTTGGTCTCTCGTGTCTGAAATTTGCTG  2960

Teqing  GATGGAACTGTTCCCATTTGGCACTGCCATAACAGTGAAACTGTGGTTTTCAGGATCATCGGGGTTGGGATCGAAGCTGA  2702
YIL25   GATGGAACTGTTCCCATTTGGCACTGCCATAACAGTGAAACTGTGGTTTTCAGGATCATCGGGGTTGGGATCGAAGCTGA  3040

Teqing  AGAAGCCATCACCAGCAGCTACTCGCGACATGTTCCCTATGCTTCTGACGGAGATCCAGCGGTTCGTTCCATCCCCGAAG  2782
YIL25   AGAAGCCATCACCAGCAGCTACTCGCGACATGTTCCCTATGCTTCTGACGGAGATCCAGCGGTTCGTTCCATCCCCGAAG  3120

Teqing  CTGCAGACTTTGCAGAGGACGTACAACTGCTTCAAGGTAAACTGGTACTTCTTGCTGAGCATTTCATTTCTAGATAGATG  2862
YIL25   CTGCAGACTTTGCAGAGGACGTACAACTGCTTCAAGGTAAACTGGTACTTCTTGCTGAGCATTTCATTTCTAGATAGATG  3200

Teqing  ATTGATTAGAATCTTGCGCTAGCTTACACAAAACAACCCCTTCGCGCTTATGATCATGCCTGTAAGTCAAAATTTAATAT  2942
YIL25   ATTGATTAGAATCTTGCGCTAGCTTACACAAAACAACCCCTTCGCGCTTATGATCATGCCTGTAAGTCAAAATTTAATAT  3280

Teqing  AAGAGTTGATTTTGTGGTTTTTT-AGTTGTTTATTTTACCGCATGAACACGTATAAAAAAGGTTTTACTATACCTTTTTT  3021
YIL25   AAGAGTTGATTTTGTGGTTTTTTTAGTTGTTTATTTTACCGCATGAACACGTATAAAAAAGGTTTTACTATACCTTTTTT  3360

Teqing  TGGTTAATAAATTGTTTGGATAAGTAAAAGCGAAACAATGGGCTGAAACACAACTGCTGATCTGCTAGGTGAATTTAGGC  3101
YIL25   TGGTTAATAAATTGTTTGGATAAGTAAAAGCGAAACAATGGGCTGAAACACAACTGCTGATCTGCTAGGTGAATTTAGGC  3440

Teqing  CCTGTTTAATTCAGCTTAGGATTATTATAATCTGGATTATTAGGATTAAGCTGAAACAAATAAGTAGATTATTATGTTAG  3181
YIL25   CCTGTTTAATTCAGCTTAGGATTATTATAATCTGGATTATTAGGATTAAGCTGAAACAAATAAGTAGATTATTATGTTAG  3520

Teqing  ATTATTATAATTTATAAGCCAGATTACTATAATATAATAATCTCCTCTAGAGGAGCTTAGATTACTATAATCTAATAATC  3261
YIL25   ATTATTATAATTTATAAGCCAGATTACTATAATATAATAATCTCCTCTAGAGGAGCTTAGATTACTATAATCTAATAATC  3600

Teqing  TCCTCTAGAGAAGCTTTTTCTAGATTATTGAGTAGCTAAAGACCCACTACCCTTAGATGCCTCTAATAATCCAGAGAAAC  3341
YIL25   TCCTCTAGAGAAGCTTTTTCTAGATTATTGAGTAGCTAAAGACCCACTACCCTTAGATGCCTCTAATAATCCAGAGAAAC  3680

Teqing  AAACAACTCGTAGCTTATTTTATGTTAGCTTATTATAATTCAGCTTAGAGTAATCTGATTTAATAATTTAGATTACAATA  3421
YIL25   AAACAACTCGTAGCTTATTTTATGTTAGCTTATTATAATTCAGCTTAGAGTAATCTGATTTAATAATTTAGATTACAATA  3760
```

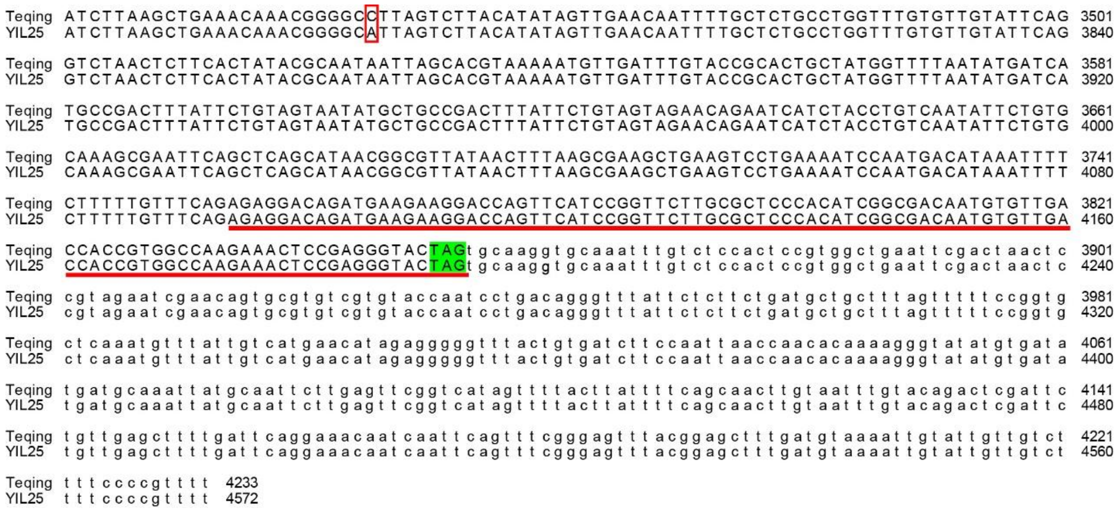

Supplementary Fig. 3. Comparison of *BOC1* sequences between Teqing and YIL25. The 337 bp deletion in the promoter region is underlined purple. The translation initiation site (ATG) and stop codon (TAG) are highlighted. The SNP variations between Teqing and YIL25 are marked by red rectangles. The 1 bp deletion is marked with a red triangle. The The 5'-UTR is underlined in rose red. The four exons of the nucleotide sequences are underlined in red. The lowercase letters indicate the 3'-UTR.

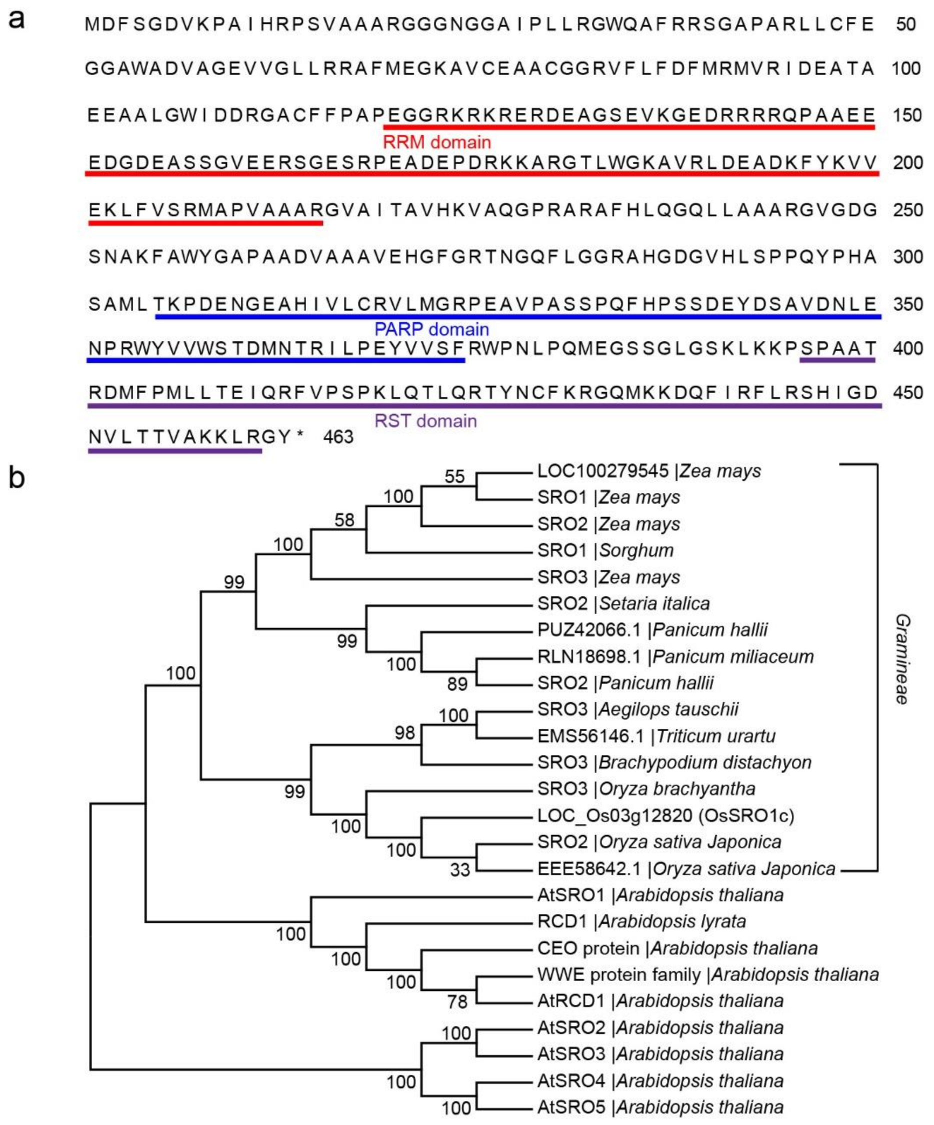

Supplementary Fig. 4. Conserved domains (a) and phylogenetic analysis (b) of BOC1 protein. The RRM domain, poly (ADP-ribose) polymerase catalytic domain (PARP), and RCD1-SRO-TAF4 (RST) domain are underlined in red, blue, and purple, respectively. The neighbor-joining tree was constructed using MEGA 7 software.

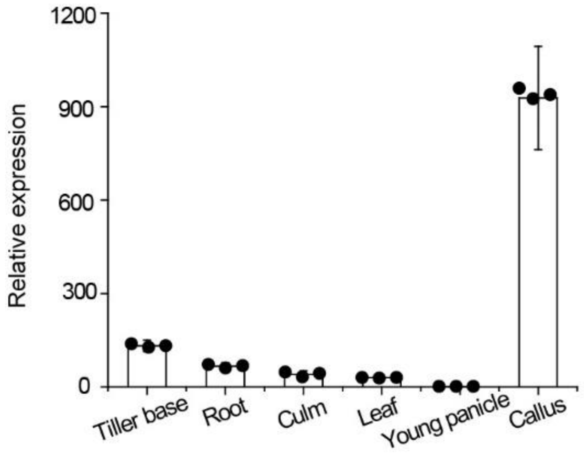

Supplementary Fig. 5. The expression pattern of *BOC1* in different organs of YIL25. Expression analysis of *BOC1* in the tiller base, root, culm, leaf, and young panicle and callus subcultured for 21 days. Data are means, with bars showing SE, *n* = 3 biological replicates. Source data are provided as a Source Data file.

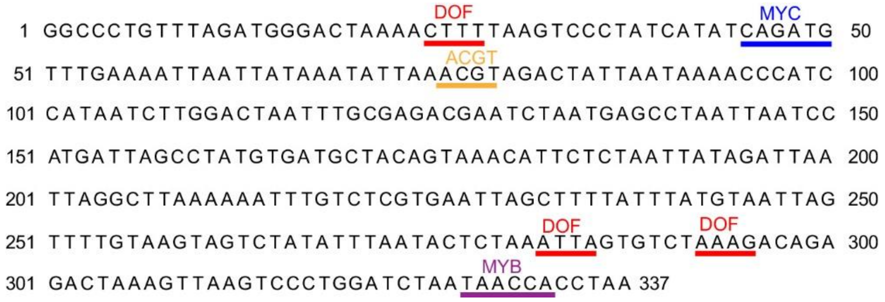

Supplementary Fig. 6. The *cis*-acting regulatory DNA elements in the 337 bp insertion associated with plant stress responses. The DOF TFBS (DOF COREZM), MYC TFBS (MYCCONSENSUSAT), ACGT TFBS (ACGTATERD1), and MYB TFBS (MYB1AT) are underlined in red, blue, brown, and purple, respectively.

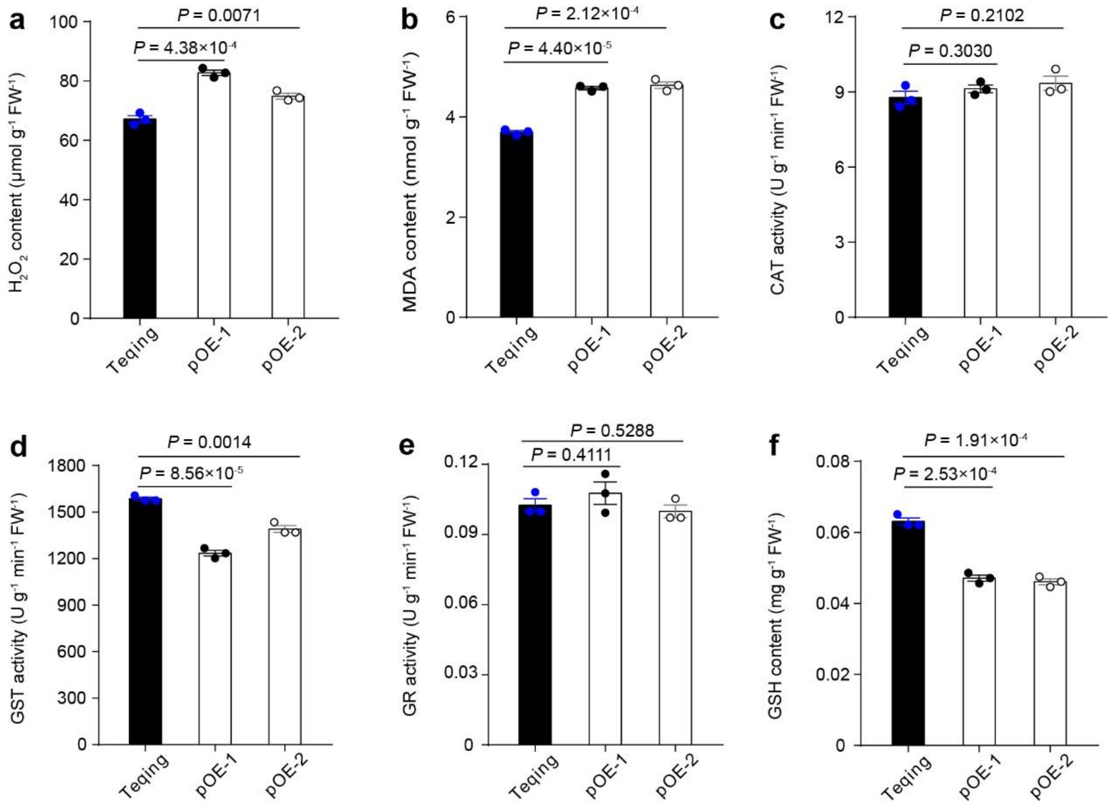

Supplementary Fig. 7. Physiological and biochemical oxidative stress indices of calli subcultured for 21 days in *BOC1* overexpression transgenic plants. (a) H_2O_2 contents in Teqing and two positive overexpression transgenic plants. (b) MDA contents. (c), (d) and (e) Assay of the antioxidant enzyme activities. CAT, catalase. GST, glutathione S-transferase. GR, glutathione reductase. (f) Antioxidant GSH contents. All values are presented as means ± SE (n = 3 biologically independent samples). P values were analyzed by two-tailed Student's t-test. Source data are provided as a Source Data file.

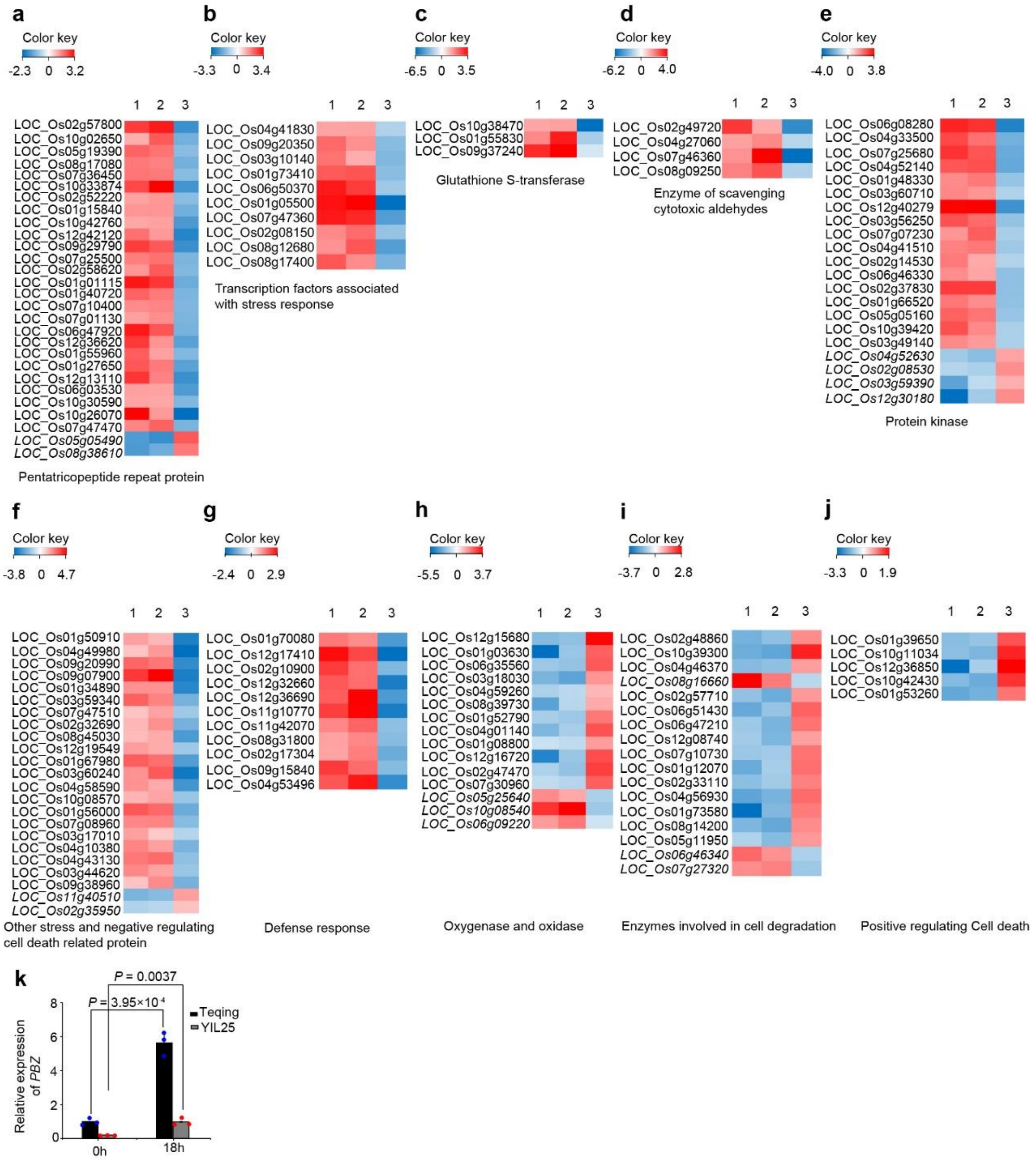

Supplementary Fig. 8. Differentially expressed genes in YIL25 vs Teqing, pCPL vs Teqing, and pOE vs Teqing via RNA-seq analysis. (a-j) 1 represents YIL25 vs Teqing. 2 represents pCPL vs Teqing. 3 represents pOE vs Teqing. (k) The relative expressions of *PBZ* by treated the callus of subculture 7d with 0.5% H_2O_2 treatment for 18h. *PBZ* represents *LOC_Os12g36850*. All values in (k) are presented as means ± SE (*n* = 3 biological replicates). *P* values were analyzed by two-tailed Student's *t*-test. Source data underlying Supplementary Figure 8k are provided as a Source Data file.

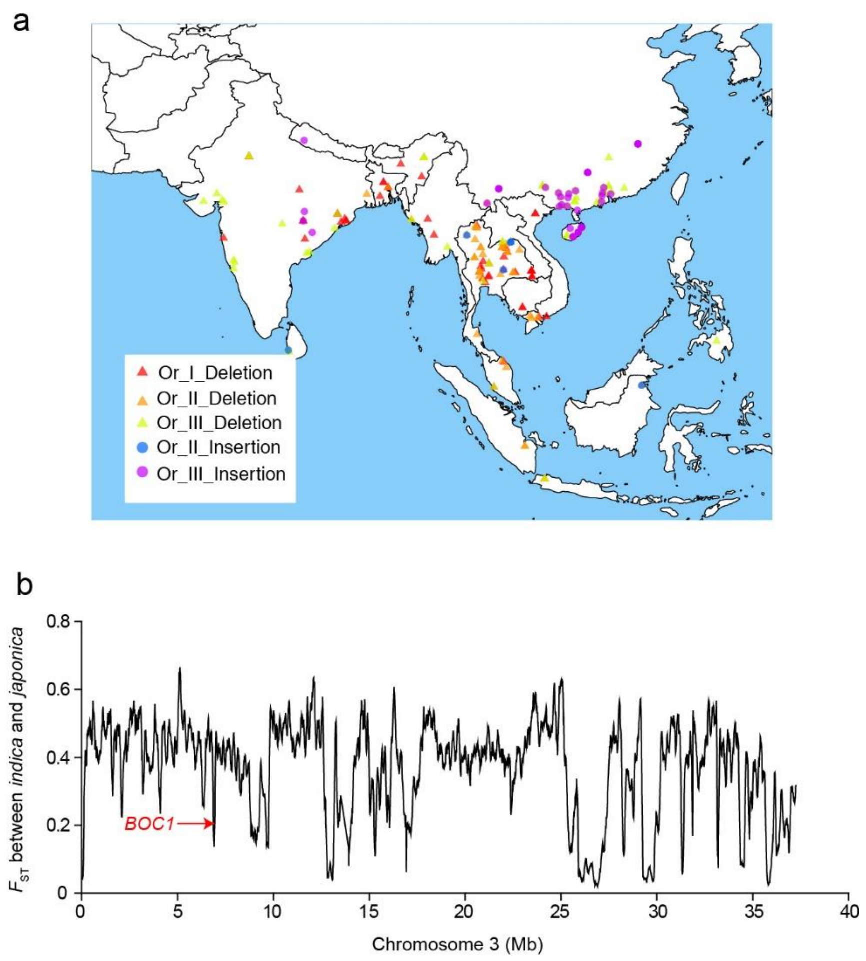

Supplementary Fig. 9. The 226 accessions of *Oryza rufipogon* classification with and without the 337 bp and the fixation index (F_{ST}) on chromosome 3 between the *indica* and *japonica* subspecies. The triangle represents the 337 bp deletion and the circle represents the 337 bp insertion. Or_I_Deletion, Or_II_Deletion, and Or_III_Deletion, represents the accessions of *Oryza rufipoon* without this 337 bp in group I, II, III, respectively. Or_II_Insertion and Or_III_Insertion represents the accessions of *Oryza rufipoon* with this insertion in group II and III, respectively. (a) was drawn by maps 3.3.0, an R package with GPL-2 license. Source data are provided as a Source Data.

Supplementary Table 1. QTL analysis using 198 F_2 individuals.

Phenotype	Locus	LOD[a]	PV[b] (%)	Add[c]	Dom[d]
Callus browning index	RM3131-RM3766	6.43	14	-5.33	-3.17

QTL was detected by single-point analysis and interval mapping. [a]: Logarithm of odds; [b]: The phenotypic variance explained by the QTL; [c]: Additive effects, negative value means YJCWR carried alleles decreasing CBI trait values. [d]: Dominant effect
