# Supplementary material for: A common wild rice-derived BOC1 allele reduces callus browning in indica rice transformation
Source: Nat Commun. 2020 Jan 23;11:443. doi: 10.1038/s41467-019-14265-0 (PMC6978460; doi:10.1038/s41467-019-14265-0)
Supplement: Supplementary file 4 — Description of Additional Supplementary Files [file 41467_2019_14265_MOESM4_ESM.docx]

**Description of Additional Supplementary Files**

File Name: Supplementary Data 1
Description: 410 DEGs up regulated in YIL25 versus Teqing, pCPL versus Teqing, and downregulated in pOE versus Teqing detected using RNA-seq.

File Name: Supplementary Data 2
Description: 290 DEGs down regulated in YIL25 versus Teqing, pCPL versus Teqing, and up regulated in pOE versus Teqing detected using RNA-seq.

File Name: Supplementary Data 3
Description: The callus browning index in the subculture 21d, BOC1 variations, and the expression level of BOC1 in 50 common wild rice.

File Name: Supplementary Data 4
Description: The callus browning index in the subculture 21d, BOC1 variations, and the expression level of BOC1 in 46 indica varieties and 28 japonica varieties.

File Name: Supplementary Data 5
Description: Primers used in this study.
