# Supplementary material for: A common wild rice-derived BOC1 allele reduces callus browning in indica rice transformation
Source: Nat Commun. 2020 Jan 23;11:443. doi: 10.1038/s41467-019-14265-0 (PMC6978460; doi:10.1038/s41467-019-14265-0)
Supplement: Supplementary file 2 — Peer Review [file 41467_2019_14265_MOESM2_ESM.pdf]

Reviewers' comments:

Reviewer #1 (Remarks to the Author):

Comments to authors
Review for "BOC1, an allele derived from common wild rice (Oryza rufipogon Griff.), reduces callus browning".
For most plants, genetic transformation is conducted through callus, followed by regeneration into plantlets. As a result, the quality of callus is critical to the success of transformation. Particularly, callus browning is detrimental to regeneration. In this study, the authors identified an allele of BOC1 from common wild rice, which mitigates callus browning. The improvement of callus quality is achieved through the insertion of a transposon, which upregulates the expression of BOC1. Overall I think this is an interest study. However I do have some comments so that the manuscript could be further improved.
1. The PIF/Harbinger transposon
The transposon identified in this study is belonging to PIF/Harbinger superfamily. However, because this is a very small element with inverted repeat, it also belongs to miniature inverted-repeat transposable element (MITEs). Due to a historical reason, MITEs in PIF/Harbinger superfamily are called Tourist elements or Tourist MITEs. This is particularly true for elements in grass family (maize, rice, wheat, etc). So it is not really wrong to call it PIF/Harbinger but it would facility the understanding to call it Tourist. My suggestion is, when you first introduce this element in the results section you may state as "it is a Tourist-like miniature inverted-repeat transposable element (MITE), which belongs to PIF/Harbinger superfamily." In the subsequent sections, you may just call it the Tourist element or the Tourist MITE. This may reduce some confusion. Please note that "Tourist" should be italicized.

Row 239 – 240 "…and that this transposable element was an important genetic resource that had been lost during the evolution from common wild rice to cultivated rice".

The language here seems to imply this element is not in cultivated rice at all but this is not true. To my knowledge, this element is very abundant (at least hundreds of copies) in the rice genome yet this particular copy at this locus is missing in cultivated rice. To minimize the confusion, please refer as "the element at this locus" or "this insertion".

Moreover, I don't think the authors really know whether this specific copy inserted prior to domestication and lost during domestication, or this element inserted after the divergence of wild rice and cultivated rice, so please consider the alternative hypothesis.

In Supplemental Figure 3, the indel position should be shifted 1 bp to the left, i.e., the element starts at "GGCC" and ends at "CACC". "TAA" is the target site duplication. Please put the extra "G" on the left side to right side of the flanking sequence. Same adjustment should be applied in Supplemental Figure 6.

2. Which group of rufipogon contains the beneficial allele?
According to Huang et al.2012 (A map of rice genome variation reveals the origin of cultivated rice), the wild rice could be divided into three groups, group I is closer to indica, group III is closer to japonica and group II is the intermedia group. Is there any way to assign the wild rice accessions (with and without the MITE insertion) in this study to the three groups to better infer the evolution pathway at this locus?
3. Figure 3
I am quite confused about the "negative" in Figure 3. It is not explained in legend or in the main text. If it refers to the segregating plants from transformation process but without the transgene, please specify it somewhere.
4. Difference between japonica and indica.

It is well known that japonica cultivars regenerate better than indica cultivars. However, since this insertion is not present in any cultivars, obvious this locus does not explain the difference between the two subspecies. This should be discussed.
5. Row 53 – 55 "However, to our knowledge, there have been no reports on the isolation of genes related to callus browning by map-based cloning in any plant species,…"
This sentence sounds like there are genes related to callus browning cloned by other methods. If that is the case, it is worth to introduce those genes here.
6. Row 138 "more than 80% sequence identity with BOC1"
Please specify the whether the sequence identity is at nucleotide or protein level.

Reviewer #2 (Remarks to the Author):

The manuscript „BOC1, an allele derived from common wild rice…"addresses BOC1, an SRO-like protein in callus browning of rice based on phenotypic mapping and map-based cloning of the BOC1 locus. The manuscript is interesting and widely well written. I only have a few concerns:

1. Regulation of aspartic proteinases and peroxidases: Not all genes are affected the same way. One gene encoding an aspartic proteinase and several genes for peroxidases are inversely regulated. Please provide more information on the two regulatory groups. Which of the genes have been associated with PCD? In which process are the others involved. In this context, quantification of apoplastic and symplastic phenol oxidases and phenol peroxidases could provide helpful information?
2. Is the effect of BOC1 on the regulation of PCD upregulated genes a direct effect? Especially downregulation of various peroxidases suggests that H2O2 / ROS metabolism is locally disturbed. Subsequent signalling might induce other peroxidases. Please provide information, which of the BOC1-disturbed genes are induced by H2O2 / ROS.
3. Relevance of l.232 – 240 in the present form unclear. Please provide information how the BOC1 locus, callus browning and the transposon are distributed. Can callus browning be evidently linked to the presence of the transposon? Is there a strict correlation between the BOC1 locus and browning? Please clarify.
4. Is there any in planta / stress tolerance effect associated with the BOC1 locus?
5. l. 33 "Introduction" missing
6. l. 326: Looks like one (introductory) sentence is missing.
7. l. 405: change order "at the wavelength of 560 nm" "based on … callus"
8. l. 410: ion: Which ion?
9. l. 404 – 411: describe method in more detail. The Fick´s laws are defined for solutions only. Here, it is used for the transport over membranes. H2O2 transport over the membrane changes with the membrane composition, e.g. with the availability of aquaporins. What is known e.g. about the H2O2 permeability of the membranes in the various cultivars?
10. l. 417: Which phosphate? The buffer is not suited for extraction. pH 6.8 does not much stabilize thiols against oxidation.
11. l. 422: provide information on the GSH concentration used
12. l. 427: give information on extraction medium / extraction procedure

Reviewer #3 (Remarks to the Author):

The manuscript is well written, original and addresses a very important topic "how to overcome recalcitrance in tissue culture for genetic improvement of crops" with a through scientific approach and detailed set of data.
Before acceptance of this article the authors should provide additional data further exploring the fact that contrasting results were obtained for pOE-BOC1-Teqing transgenic lines expressing BOC1 under

the constitutive ubiquitin promoter and pCPL-BOC1-Teqing transgenic lines expressing BOC1 under native regulatory elements from line YIL25 with additional transgenic lines. An important question that need to be answered here is the line to line variation with these constructs for BOC1 expression level and callus browning. The data should be presented in one figure or table (combining figure 3 and suppl. Figure 7) for a better side by side comparison. Currently only 2 lines are presented for pOE-BOC1-Teqing and two lines for pCPL-BOC1-Teqing and a larger number of lines (e.g. 10 for each construct) differing in the expression level should be included to demonstrate the potential of this approach to suppress callus browning and to clarify which expression level is desirable for suppression of callus browning or exacerbates callus browning. It would also be desirable to include RNAseq data from pOE-BOC1-Teqing and pCPL-BOC1-Teqing transgenic lines (currently RNAseq data only include Teqing and YIL25).
A description of the experiment that resulted in the data for fig. 5 is missing in the material and methods. It is not clear how many calli were used for transformation in each experiment. Three experiments (for data in fig. 5) seems a small number considering that typically large variation is found in transformation efficiency from experiment to experiment. Therefore, at least 10 independent experiments should be presented for figure 5.

Minor aspects that can be addressed to improve the manuscript:
Results:
Line 90: Rephrase "the cells of Teqing appeared introcession"
Line 94-95: "First, we measured the CBI on the seeds of the 198 individual plants." Please clarify if the CBI was assessed on the seeds or on seed derived callus. Also include data in supplementary file or cite paper if published elsewhere.
Line 123-124: Replace "containing the promoter and open reading frame of LOC_Os03g12820" with "containing the promoter, open reading frame and 3' UTR of LOC_Os03g12820"

Line 130: Rephrase to "results suggest that LOC_Oso3g12820 is BOC1 regulates callus browning in rice."

Line 153-154: Rephrase to " which indicated that the expression of BOC1 in YIL25 calli was higher than in Teqing calli (Fig. 4b)."

Line 180: Rephrase to "lines were worse"

Material and Methods:
Line 332-333. Transformation efficiency is defined as "(number of positive plants for independent transgenic events/number of transferred calli) ×100%" and should be: "(number of independent transgenic events regenerating plants/number of transferred calli) ×100%"
Line 684: Figure legend for Fig. 5 "Data were repeated three times" Do the authors intend to state "means represent data from three independent experiments"?
Figure legends for Figure 3 and supplementary figure 7: For clarity the genetic background of the "negative transgenic line" should be described.

Dear Reviewers,

Thank you very much for your excellent comments on our manuscript "***BOC1*, an allele derived from common wild rice (*Oryza rufipogon* Griff.), reduces callus browning**" (**ID: NCOMMS-19-09797**). Those comments are all valuable and very helpful for revising and improving our paper, as well as the important guiding significance to our study. We have carried out additional experiments, analyzed the relevant data and revised the manuscript accordingly. We used red color to mark the modified words and sentences in main text and figure legends. The detailed responses are shown below. We would be grateful if you can kindly be satisfied with the revised manuscript.

Thank you again for your time and efforts in taking care of our manuscript.

Sincerely,

Yongcai Fu, Ph.D., Professor
Department of Plant Genetics and Breeding
China Agricultural University, Beijing 100193, China
Tel: 86-10-62733507
E-mail: yongcaifu@cau.edu.cn

Reponses to reviewer #1's comments:

Review for "*BOC1*, an allele derived from common wild rice (*Oryza rufipogon* Griff.), reduces callus browning". For most plants, genetic transformation is conducted through callus, followed by regeneration into plantlets. As a result, the quality of callus is critical to the success of transformation. Particularly, callus browning is detrimental to regeneration. In this study, the authors identified an allele of *BOC1* from common wild rice, which mitigates callus browning. The improvement of callus quality is achieved through the insertion of a transposon, which upregulates the expression of *BOC1*. Overall I think this is an interest study. However I do have some comments so that the manuscript could be further improved.

Response: Thank you very much for your positive comments. These comments are very helpful for revising our manuscript. Following your suggestion, we have carefully modified the manuscript. We also added relevant experiments and analyzed the data. Thank you again for your useful suggestions on our manuscript.

Reviewer #1's question 1 **(*R1Q1*):** The PIF/Harbinger transposon

The transposon identified in this study is belonging to PIF/Harbinger superfamily. However, because this is a very small element with inverted repeat, it also belongs to miniature inverted-repeat transposable element (MITEs). Due to a historical reason, MITEs in PIF/Harbinger superfamily are called Tourist elements or Tourist MITEs. This is particularly true for elements in grass family (maize, rice, wheat, etc). So it is not really wrong to call it PIF/Harbinger but it would facility the understanding to call it Tourist. My suggestion is, when you first introduce this element in the results section you may state as "it is a Tourist-like miniature inverted-repeat transposable element (MITE), which belongs to PIF/Harbinger superfamily." In the subsequent sections, you may just call it the Tourist element or the Tourist MITE. This may reduce some confusion. Please note that "Tourist" should be italicized.

Row 239 – 240 "…and that this transposable element was an important genetic resource that had been lost during the evolution from common wild rice to cultivated rice".

The language here seems to imply this element is not in cultivated rice at all but this is not true. To my knowledge, this element is very abundant (at least hundreds of copies) in the rice genome yet this particular copy at this locus is missing in cultivated rice. To minimize the confusion, please refer as "the element at this locus" or "this insertion".

Moreover, I don't think the authors really know whether this specific copy inserted prior to domestication and lost during domestication, or this element inserted after the divergence of wild rice and cultivated rice, so please consider the alternative hypothesis.

In Supplemental Figure 3, the indel position should be shifted 1 bp to the left, i.e., the element starts at "GGCC" and ends at "CACC". "TAA" is the target site duplication. Please put the extra "G" on the left side to right side of the flanking sequence. Same adjustment should be applied in Supplemental Figure 6.

Response: Thank you very much for your suggestion.

(1) Following your suggestion, we have added "it is a *Tourist*-like miniature inverted-repeat transposable element (MITE), which belongs to PIF/Harbinger superfamily" (line 184-186). In the subsequent sections, we call it "*Tourist* MITE".

(2) We agree with your suggestion. The description in the previous manuscript made some confusion. We have revised the description to "the element at this locus" or "this insertion" (line 290, 298, 300, 367).

(3) Indeed, we don't know whether this specific copy inserted prior to domestication and lost during domestication, or this element inserted after the divergence of wild rice and cultivated rice, so we crossed out this sentence.

(4) According to your suggestion, we put the extra "G" on the left side to the

right side of the flanking sequence and highlighted in red (Supplementary Fig. 3).

Thank you again for your helpful comments!

R1Q2: Which group of rufipogon contains the beneficial allele?

According to Huang et al.2012 (A map of rice genome variation reveals the origin of cultivated rice), the wild rice could be divided into three groups, group I is closer to indica, group III is closer to japonica and group II is the intermedia group. Is there any way to assign the wild rice accessions (with and without the MITE insertion) in this study to the three groups to better infer the evolution pathway at this locus?

Response: Following your suggestion, we downloaded the genome resequencing data of 446 accessions of *Oryza rufipogon* from the previous report[1]. We recovered the reads mapped to the 337 bp flanking sequence in all accessions. The result showed that a total of 226 accessions were detected in this site (48 accessions with this MITE insertion and 178 accessions without this insertion, **Fig. 1**). Forty-one of the 48 accessions including this insertion were in group III and 7 were in group II (**Fig. 1**). Nevertheless, the 178 accessions without this MITE insertion distributed in all the three groups (65 in group I; 49 in group II; 64 in group III) (**Fig .1**). We accepted your valuable suggestion, and had added the result in our revised manuscript (line 294-302).

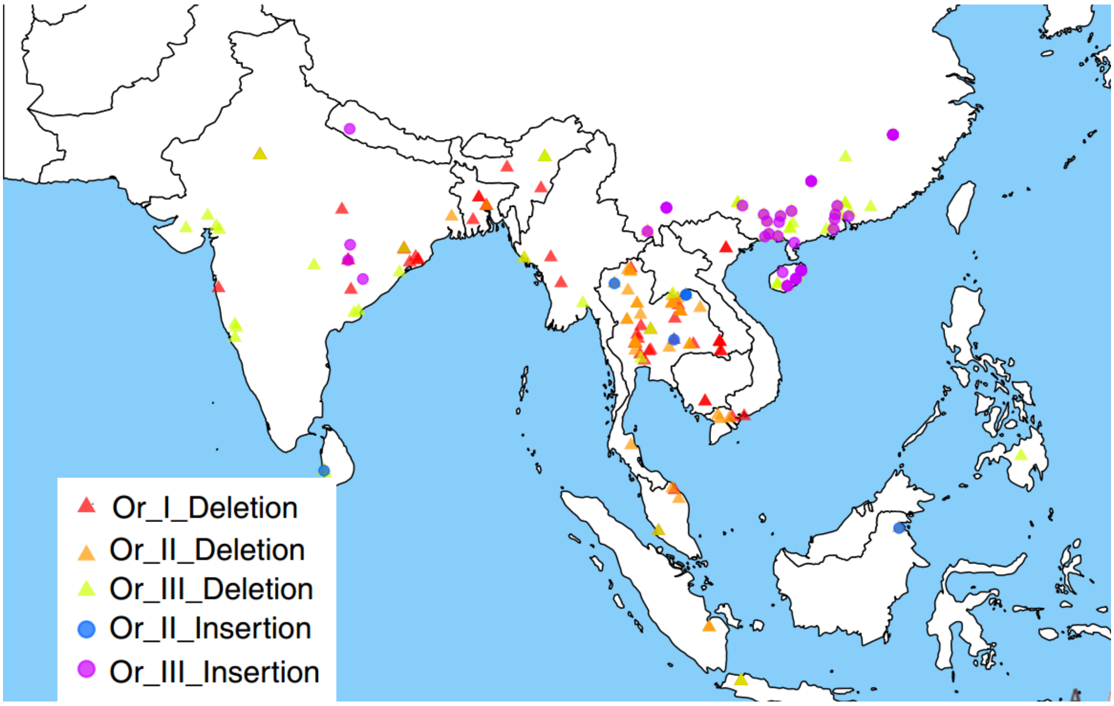

Fig. 1 The 226 accessions of *Oryza rufipogon* classification with and without the 337 bp. The triangle represents the 337 bp deletion and the circle represents the 337 bp insertion. Or_I_Deletion, Or_II_Deletion, and Or_III_Deletion, represents the accessions of *Oryza rufipoon* without this 337 bp in group I, II, III, respectively. Or_II_Insertion and Or_III_Insertion represents the accessions of *Oryza rufipoon* with this insertion in group II and III, respectively.

1. Huang, X.H. et al. A map of rice genome variation reveals the origin of cultivated rice. *Nature* **490**, 497-501 (2012).

R1Q3: Figure 3

I am quite confused about the "negative" in Figure 3. It is not explained in legend or in the main text. If it refers to the segregating plants from transformation process but without the transgene, please specify it somewhere.

Response: Thank you for your helpful comment, it's our negligence. Yes, "negative" refers to the segregating plants from transformation process but without the transgene. In response to your suggestion, we had specified it in the Figure 3 legends of our manuscript. Thank you!

R1Q4: Difference between japonica and indica.

It is well known that japonica cultivars regenerate better than indica cultivars.

However, since this insertion is not present in any cultivars, obvious this locus does not explain the difference between the two subspecies. This should be discussed.

Response: Thank you very much for your suggestion. We analyzed the fixation index (F_{ST}) on chromosome 3 between different subspecies using previously sequenced rice genomes[1]. The result showed that the F_{ST} level in the *BOC1* gene was 0.21 (**Fig. 2**), indicating that no significant differences might exist in *BOC1* between the *indica* and *japonica* subspecies. Therefore, we proposed that the effect of *BOC1* on callus browning has nothing to do with the differences of *indica* and *japonica* subspecies. We also added this result to our modified manuscript (line 315-320).

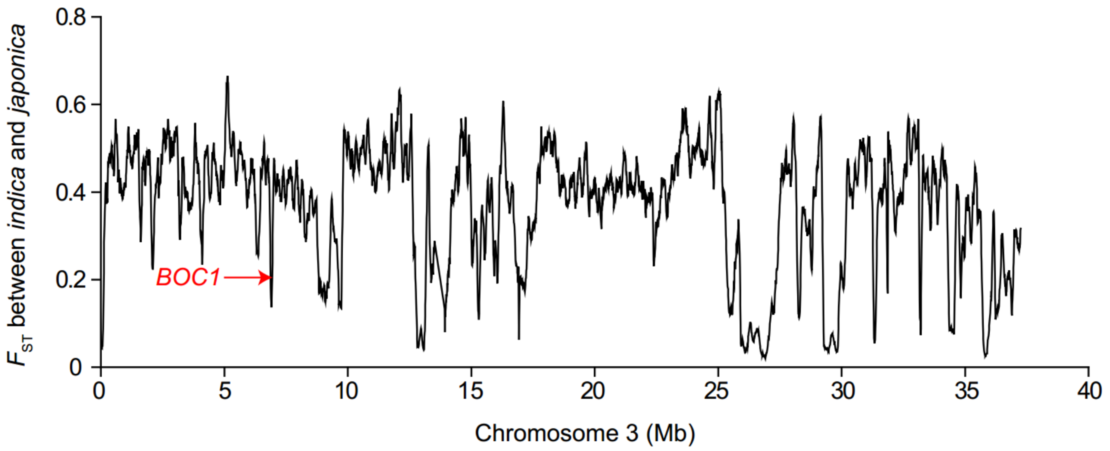

Fig .2 the fixation index (FST) on chromosome 3 between the *indica* and *japonica* subspecies.

1. Huang, X.H. et al. A map of rice genome variation reveals the origin of cultivated rice. *Nature* **490**, 497-501 (2012).

R1Q5: Row 53 – 55 "However, to our knowledge, there have been no reports on the isolation of genes related to callus browning by map-based cloning in any plant species,…"

This sentence sounds like there are genes related to callus browning cloned by other methods. If that is the case, it is worth to introduce those genes here.

Response: Thank you for your helpful comment. So far, no genes related to callus browning have been isolated by any strategy. We deleted "by map-based" (line 56-59).

R1Q6: Row 138 “more than 80% sequence identity with BOC1”

Please specify the whether the sequence identity is at nucleotide or protein level.

Response: Thank you very much for your suggestion. We revised the “more than 80% sequence identity with BOC1 at nucleotide level” in our manuscript of line 153.

Reponses to reviewer #2's comments:

The manuscript „BOC1, an allele derived from common wild rice…"addresses BOC1, an SRO-like protein in callus browning of rice based on phenotypic mapping and map-based cloning of the BOC1 locus. The manuscript is interesting and widely well written. I only have a few concerns:

Response: Thank you very much for your excellent and detailed suggestions. These suggestions will play an important role in improving our manuscript. According to your suggestions, we revised the manuscript and also added the relevant experiments and analyzed the data.

Reviewer #2's question 1 **(*R2Q1*):** Regulation of aspartic proteinases and peroxidases: Not all genes are affected the same way. One gene encoding an aspartic proteinase and several genes for peroxidases are inversely regulated. Please provide more information on the two regulatory groups. Which of the genes have been associated with PCD? In which process are the others involved. In this context, quantification of apoplastic and symplastic phenol oxidases and phenol peroxidases could provide helpful information?

Response: Thank you very much for your helpful comments. Yes, actually, our previous differential expression analysis was insufficient and deficient. In our previous study, only differential expression of Teqing and YIL25 was analyzed. However, YIL25 contained three YJCWR chromosomal segments on the short arm of chromosomes 2, 3, and 5 and other genes were also present in the same YJCWR chromosomal segments, which affected the accuracy of the results. Following your suggestion, in our revised manuscript, we added RNA-seq experiments using callus that had been subcultured for 21 days from Teqing and the complementation line pCPL-*BOC1*-Teqing (pCPL), and Teqing and the overexpression line pOE-*BOC1*-Teqing (pOE). We identified a total of 700 differentially expressed genes (DEGs), as well as 410 DEGs upregulated in the callus of YIL25 versus Teqing, pCPL versus Teqing and downregulated in pOE versus Teqing (UUD, **Fig. 7a**, **Supplementary Table. 2**). Besides, 290 DEGs downregulated in the callus of YIL25 versus Teqing and pCPL versus Teqing

and upregulated in pOE versus Teqing (DDU, fold change ≥ 2, FDR < 0.001) (**Fig. 7b**, **Supplementary Table. 3**). A large number of differentially expressed genes related to ROS production, stress response and cell death were found in the overlap of UUD or DDU. These results supported that appropriate improving expression level of *BOC1* was beneficial for the cells to be in redox state, prevented various stress and then inhibited cell death, while the overexpression lines on the contrary. These results were described in the revised manuscript (line 233-275).

In addition, many reports suggested that callus browning is caused by increased polyphenol oxidase (PPO) and peroxidases (POD) activity. Following your suggestion, we measured the PPO activity of Teqing and YIL25 in different development of tissue culture (**Fig. 1a**) and the POD containing phenol peroxidases activity of Teqing and complementary transgenic lines **(Fig. 1b)**. However, we found irregular results, which showed that the relationship of *BOC1* and callus browning potentially may be mainly through regulating cell death.

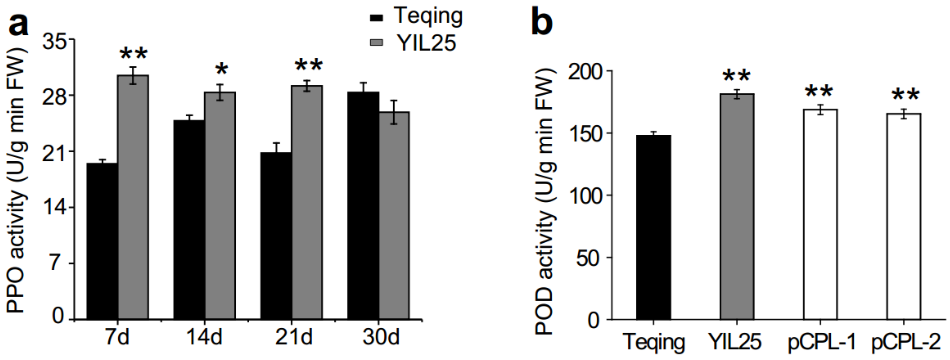

Fig.1 PPO and POD activities.

(a) PPO activities of Teqing and YIL25 in different development of tissue culture. (b) POD activities of Teqing and complementary transgenic lines.

R2Q2**:** Is the effect of BOC1 on the regulation of PCD upregulated genes a direct effect? Especially downregulation of various peroxidases suggests that H2O2 / ROS metabolism is locally disturbed. Subsequent signalling might induce other peroxidases. Please provide information, which of the BOC1-

disturbed genes are induced by H2O2 / ROS.

Response: Thank you very much for your beneficial comments. Your suggestions are very helpful for us to analyze the results of RNA-seq data.

In our new RNAseq analysis results, many upregulated genes associated with cell death were disturbed in YIL25 and *BOC1* transgenic complementary lines, for instance, aspartic proteinase, peptidase, nuclease and enzymes involved in cell wall degradation (polygalacturonase, endoglucanase and glycosyl hydrolases). Besides, some genes positively regulating senescence process were present, for example, *LOC_Os01g53260* encodes *WRKY* transcription factor *OsWRKY23*, and *OsWRKY23*, positively regulating senescence process[1], was in DEGs of DDU. *LOC_Os12g36850* encodes pathogenesis-related bet v I family protein, similar to probenazole-inducible protein PBZ1 and PBZ1 induces cell death not only in rice, but also in tobacco and *Arabidopsis* via its RNase activity inside the cell[2]. We added this result to our revised manuscript (line 261-271)

It has been reported that OsSRO1c, an allele of BOC1, has no transcriptional activity[3], and we verified this results by experiment **(Fig.2)**. There has been reports that OsSRO1c interacts with a large number of diverse proteins[3]. OsSRO1c-interacting proteins (SRIPs) were isolated by yeast two-hybrid screening and BiFC assay in living rice cells and the results confirmed that OsSRO1c interacts with OsDREB2B, OsNAC8, and UBA[3]. In addition, OsSRO1c interacts with *Arabidopsis* transcription factors, for example, AP2–EREBP, bHLH, bZIP, C2H2 zinc finger, MYB, and WRKY[4].

More genes associated with peroxidases (POD) were detected in RNA-seq analysis between YIL25 and Teqing of our previous manuscript. It has been reported that POD catalyzes the oxidation of phenols and affects the browning of callus[5]. Moreover, as your suggestion, POD also metabolizes H_2O_2 / ROS. Therefore, the function of POD involved in regulating callus browning was complicated, which may be in different intracellular environments and act as local disturbance. In our new RNAseq analysis results, only one peroxidase

precursor was found, which downregulated in the callus of YIL25 versus Teqing and pCPL versus Teqing and upregulated in pOE versus Teqing. Those results indicated that the conclusion of original manuscript about peroxidase may be affected by other genes of introgression fragments.

Meanwhile, we treated the callus of subculture 7d with 0.5% H_2O_2 for 18h and detected the *PBZ* (*LOC_Os12g36850*) was upregulated induced by H_2O_2 **(Supplementary Fig. 8k)**. We put this result into our modified manuscript (line 271-273), which provides more evidence for our conclusion.

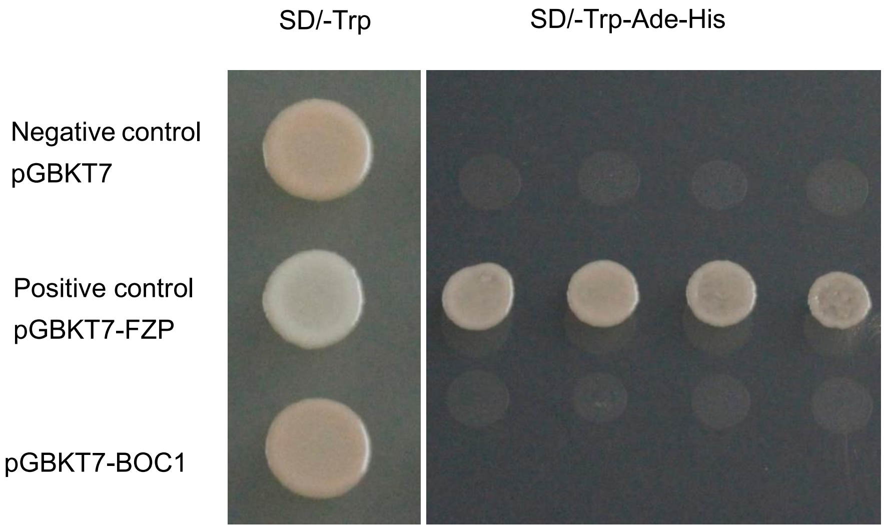

Fig. 2 The detection of BOC1 transcriptional activity.

1. Jing, S.J, Zhou, X., Song, Y. & Yu, D.Q. Heterologous expression of *OsWRKY23* gene enhances pathogen defense and dark-induced leaf senescence in *Arabidopsis*. *Plant Growth Regul.* 58, 181-190 (2009).
2. Kim, S.G. et al. The RNase activity of rice probenazole-induced protein1 (PBZ1) plays a key role in cell death in plants. *Mol. Cells* 31, 25-31 (2011).
3. You, J. et al. The SNAC1-targeted gene *OsSRO1c* modulates stomatal closure and oxidative stress tolerance by regulating hydrogen peroxide in rice. *J. Exp. Bot.* **64**, 569–583 (2013).
4. You et al. A special member of the rice SRO family, OsSRO1c, mediates responses to multiple abiotic stresses through interaction with various transcription factors. *Plant Mol. Biol.* **84**, 693-705 (2014).

5. Laukkanen, H., Häggman, H., Kontunen-Soppela, S. & Hohrola, A.J. Tissue browning of *in vitro* cultures of scots pine: role of peroxidase and polyphenol oxidase. *Physiol*. *Plant*. **106**, 337–343 (1999).

***R2Q3*:** Relevance of l.232 – 240 in the present form unclear. Please provide information how the BOC1 locus, callus browning and the transposon are distributed. Can callus browning be evidently linked to the presence of the transposon? Is there a strict correlation between the BOC1 locus and browning? Please clarify.

Response: Thank you for your excellent suggestions.

In our original manuscript, some cultivars has no seeds or not enough to repeat three times or be contaminated in tissue culture, therefore, we screened total of 74 Asian rice cultivars (28 *japonica* and 46 *indica* cultivars). Moreover, it is very difficult to collect the seeds of accessions of *O. rufipogon* and only 6 accessions were enough to analyze the callus browning index, so we recollected the additional seeds of 44 accessions of *O. rufipogon* from Rice Research Institute of Guangdong Academy of Agricultural Sciences in China and Rice Research Institute of Guangxi Academy of Agricultural Sciences in China and also added the Xiaoyang Zhu and Xin Ma to help us analyze the data. Therefore, we added Xiaoyang Zhu, Xin Ma, Chen Li and Yuntao Liang to “authors” column. The results are as follows:

To investigate the relationships between the mutations in the *BOC1* locus and callus browning, we sequenced a ~4.6 kb genomic fragment covering the entire *BOC1* gene (2,740 bp), a 1,452 bp 5′-flanking region, and a 380 bp 3′-flanking region, from 50 accessions of *O. rufipogon* **(Supplementary Table. 4)** and 74 Asian rice cultivars **(**28 *japonica* and 46 *indica* cultivars, **Supplementary Table. 5).** Nucleotide alignment showed that only 19 accessions of *O. rufipogon* exhibited the presence of the *Tourist* MITE **(Supplementary Table. 4)**, however, the element at this locus of all the sequenced Asian rice cultivars was deleted **(Supplementary Table. 5)**. Therefore, an association test of the accessions of *O. rufipogon* and the Asian

rice cultivars between callus browning of subculture 21d and sequence variations were analyzed, respectively. The association test of the common wild rice showed that the strongest association signals were present at the *Tourist* MITE and 4 SNPs **(Fig. 8a)**. Furthermore, we investigated the CBI of the 50 common wild rice and found that the CBI of 19 accessions with the *Tourist* MITE was lower than 31 accessions without this insertion **(Fig. 8b)**. Moreover, we tested the *BOC1* gene expression of the 19 accessions with the *Tourist* MITE and 31 accessions without the *Tourist* MITE, and the results showed that the expression levels of *BOC1* in these varieties with the *Tourist* MITE were higher than that without the *Tourist* MITE **(Fig. 8c, Supplementary Table. 4)**. In addition, we downloaded the genome resequencing data of 446 accessions of *Oryza rufipogon* from the previous report[1] and recovered the reads mapped to the *Tourist* MITE flanking sequence in all accessions. The result showed that a total of 226 accessions were detected in this site (48 accessions with this MITE insertion and 178 accessions without this insertion). Forty-one of the 48 accessions including this insertion were in group III (closer to *japonica*) and 7 were in group II (the intermedia group between *indica* and *japonica*) **(Supplementary Fig. 8a)**. Nevertheless, the 178 accessions without this MITE insertion were distributed in all the three groups (65 in group I (closer to *indica*); 49 in group II; 64 in group III) **(Supplementary Fig. 8a)**.

Nucleotide alignment of the 74 Asian rice cultivars revealed 9 Indels and 27 SNPs **(Fig. 8d, Supplementary Table. 4)**. To ascertain whether other locus of *BOC1* was responsible for callus browning in Asian rice cultivars, firstly, an association test of the 74 Asian rice cultivars between callus browning and sequence variations were studied. The result indicated that the Indel8 (-433, T indel) in the promoter of *BOC1* was strongest signals, however this site was no polymorphism in the 50 accessions of *O. rufipogon* **(Fig. 8d, Supplementary Table. 4, 5)**. We further founded the CBI in 15 varieties with T insertion in the promoter of *BOC1* was lower than that in 59 varieties with T

deletion **(Fig. 8e, Supplementary Table. 5)**. We also analyzed the expression levels in these varieties and the result showed that the expression levels in 15 varieties with T were higher than that in 59 varieties without T **(Fig. 8f, Supplementary Table. 5)**. These results indicated that the structure variation of *BOC1* promoter influenced the expression of *BOC1* and further affected callus browning. We added these results to the revised manuscript (line 277-314).

1. Huang, X.H. et al. A map of rice genome variation reveals the origin of cultivated rice. *Nature* **490**, 497-501 (2012).

4. Is there any in planta / stress tolerance effect associated with the BOC1 locus?

Response: Thank you very much for your helpful comment.

OsSRO1c (*Similar to Radical-Induced Cell Death One 1c*), which is encoded by *BOC1* in rice, and *AtRCD1* (*Radical-induced Cell Death1*) in *Arabidopsis* received its name due to the ozone hypersensitive phenotype observed in plant knock-outs to this gene[1]. *OsSRO1c*, an allele of *BOC1*, was responsive to multiple stresses. *OsSRO1c* had dual roles in drought and oxidative stress tolerance of rice. The *ossro1c-1* mutant was sensitive to drought stress. Interestingly, *OsSRO1c*-overexpressing rice showed increased sensitivity to oxidative stress. However, plants overexpressing *SNAC1*, a NAC transcription factor that directly regulates *OsSRO1c*, had drought resistance. That had the potential to the expression level of *OsSRO1c* in *SNAC1*-overexpressing plants is not as high as in *OsSRO1c*-overexpressing plants and therefore no significant oxidative stress occurred by overexpression of SNAC1[2]. In addition, the *ossro1c-1* mutant showed resistance to oxidative stress, however, the *ossro1c-1* mutant and artificial microRNA-*OsSRO1c* transgenic rice were significantly impaired in cold tolerance[3].

The metabolic pathways between the callus and the planta were different and also the expression in planta and the response to stress of *BOC1* may be differences. Study showed that the transcriptome and how it responds to a

stress differs between tissues or organs of the same organism[1]. Besides, different materials in research may get the different results. This question "Is there any in planta / stress tolerance effect associated with the *BOC1* locus" you pointed out is what we focus on and think about deeply and we will investigate and analyze them in detail and separately in our future studies, yet, this may take a long time.

1. de Abreu Neto, J.B. & Frei, M. Microarray meta-analysis focused on the response of genes involved in redox homeostasis to diverse abiotic stresses in rice. *Front*. *Plant Sci*. **6** (2016).
2. You, J. et al. The SNAC1-targeted gene *OsSRO1c* modulates stomatal closure and oxidative stress tolerance by regulating hydrogen peroxide in rice. *J. Exp. Bot.* **64**, 569–583 (2013).
3. You et al. A special member of the rice SRO family, OsSRO1c, mediates responses to multiple abiotic stresses through interaction with various transcription factors. *Plant Mol. Biol.* **84**, 693-705 (2014).

5. l. 33 "Introduction" missing

Response: Following the guide for submission to *Nature Communications*, the article avoids "Introduction" as a heading. So we delete the "Introduction". Thank you for reminding us!

6. l. 326: Looks like one (introductory) sentence is missing.

Response: According your comment, we added one (introductory) sentence "The tendency of callus browning, selection frequency of *Hyg*-resistant calli and transformation efficiency were calculated according to the following formula" on the revised manuscript of line 401-403. Thank you!

7. l. 405: change order "at the wavelength of 560 nm" "based on … callus"

Response: Thank you for your helpful suggestion. We have followed your suggestion and change order "analyzed based on the fresh weight of callus at the wavelength of 560 nm" to "analyzed at the wavelength of 560 nm based on the fresh weight of callus" (line 484).

8. l. 410: ion: Which ion?

Response: Thank you very much for pointing this out, it's our negligence. We had modified the "ion" to "analyte H_2O_2" on the revised manuscript of line 497.

9. l. 404 – 411: describe method in more detail. The Fick´s laws are defined for solutions only. Here, it is used for the transport over membranes. H2O2 transport over the membrane changes with the membrane composition, e.g. with the availability of aquaporins. What is known e.g. about the H2O2 permeability of the membranes in the various cultivars?

Response: Thank you for your suggestion! The H_2O_2 fluxes obtained via Non-invasive Micro-test Technology (NMT) revealed two information: (1) how many H_2O_2 and how fast these H_2O_2 are moving; (2) the direction of their motions, after they left the membrane diffusing into the surrounding liquid media, or before they entered into the cell. The test combined with drugs by which can either block or enhance the activities of these aquaporins, we may get information about their roles played by them in this process. The detailed method was as follows: we screened the callus of subculture 21d and fixed the calli on the bottom of the petri with filter paper strip and resin block. Add the measuring solution to the petri dish, soak the callus and leave it for about 20 minutes. Then discard the measuring solution and add 5~10 ml of fresh measuring solution. The measuring solution formulation: 0.1 mM KCl, 0.1 mM $CaCl_2$, 0.1 mM $MgCl_2$, 0.5 mM NaCl, 0.3 mM MES, 0.2 mM Na_2SO4, pH 6.5. After that, the H_2O_2 flux microsensor was placed about 10 μm distance from the calli, measured 10 min of each site and repeated 4 times. In the end, get the flux data using the imFluxes V2.0 software (YoungerUSA LLC, Amherst, MA 01002, USA), and the positive value/ the negative value represents efflux and influx, respectively. We added the measuring method to our modified manuscript (line 487-496).

10. l. 417: Which phosphate? The buffer is not suited for extraction. pH 6.8 does not much stabilize thiols against oxidation.

Response: Thank you for your comment, it's our negligence. The buffer we used is 0.1 M NaH_2PO_4 (pH 7.7), not pH 6.8. The detection of GSH content was carried out in Shanghai Sanshu Biotechnology Co., LTD (Shanghai, China). We added "0.1 M NaH_2PO_4 (pH 7.7)" to our modified manuscript (line 504-506).Thank you very much for your reminding.

11. l. 422: provide information on the GSH concentration used

Response: The concentration of oxidized glutathione we used was 10 mM. We added the "The reaction mixture of GR contained 1.0 ml of 0.1 M potassium phosphate buffer (pH 8.0) including 0.5 mM EDTA, 0.5 mM $MgCl_2$,1 mM NADPH, 10 mM oxidized glutathione (GSSG), and 0.1 ml enzyme extract" to our revised manuscript (line 513-517). Thank you!

12. l. 427: give information on extraction medium / extraction procedure

Response: Thank you for your comments. We added the information on extraction medium / extraction procedure "The calli were ground into powder in liquid nitrogen in mortar and transferred each sample (0.05 g) to 1.5 ml screw-cap tubes. We then added 50 µl internal standards and 500 µl extraction solvent (2-propanol: H_2O: HCl = 2： 1： 0.002) to each tube and on a shaker at a speed of 900 rpm for 30 min at 4°C. Each sample was added 1 ml trichloromethane, shook for 30 min, centrifuged at 14,000 rpm for 5 min at 4°C and then two phases were formed. Then we transferred 1.2 ml solvent from the lower phase, drought the solvent using a nitrogen evaporator and added 0.1 ml methanol to redissolve and quantitative analysis of endogenous salicylic acid from the sample was measured by high-performance liquid chromatography-mass spectrometry (HPLC-MS/MS)" to our modified manuscript (line 519-527). Thank you!

Reponses to reviewer #3's comments:

The manuscript is well written, original and addresses a very important topic "how to overcome recalcitrance in tissue culture for genetic improvement of crops" with a through scientific approach and detailed set of data.

Response: Thank you very much for your positive comments. Those comments are all valuable and very helpful for modifying and improving our manuscript, and also the important guiding significance to our report. We have added the relevant experiments, analyzed the relevant data and revised the manuscript according your suggestions.

***Reviewer #3's question 1* (*R3Q1*):** Before acceptance of this article the authors should provide additional data further exploring the fact that contrasting results were obtained for pOE-BOC1-Teqing transgenic lines expressing BOC1 under the constitutive ubiquitin promoter and pCPL-BOC1-Teqing transgenic lines expressing BOC1 under native regulatory elements from line YIL25 with additional transgenic lines. An important question that need to be answered here is the line to line variation with these constructs for BOC1 expression level and callus browning. The data should be presented in one figure or table (combining figure 3 and suppl. Figure 7) for a better side by side comparison. Currently only 2 lines are presented for pOE-BOC1-Teqing and two lines for pCPL-BOC1-Teqing and a larger number of lines (e.g. 10 for each construct) differing in the expression level should be included to demonstrate the potential of this approach to suppress callus browning and to clarify which expression level is desirable for suppression of callus browning or exacerbates callus browning. It would also be desirable to include RNAseq data from pOE-BOC1-Teqing and pCPL-BOC1-Teqing transgenic lines (currently RNAseq data only include Teqing and YIL25).

Response: Thank you very much for your excellent and helpful suggestions. We accepted your valuable suggestions, and had added the relevant results in our revised manuscript.

(1) In response to your suggestion, we compared the line to line variations with

pRi-*BOC1*-YIL25, pCPL-*BOC1*-Teqing and pOE-*BOC1*-Teqing for *BOC1* expression level and callus browning. Based on the expression of Teqing or Negative (Teqing), we analyzed the line to line variations with these constructs. The result indicated that the positive pRi-*BOC1*-YIL25 lines had serious callus browning compared to the negative plants, since when the expression of *LOC_Os03g12820* in the pRi-*BOC1*-YIL25 positive transgenic lines was reduced to the level of Teqing. The callus browning index of pCPL-*BOC1*-Teqing was reduced in these positive lines since when the relative expression level of *LOC_Os03g12820* was higher than 1.8 times compared with negative (Teqing), however, when the *BOC1* expression levels in the pOE-*BOC1*-Teqing positive transgenic lines were higher than 8.8 times compared with the negative (Teqing), the callus browning phenotypes of the tested pOE-*BOC1*-Teqing positive lines were worse than the negative plants (Teqing). Following your suggestion, we put the expression and CBI of all lines to one figure (**Fig. 1**). However, the expressions of pOE-*BOC1*-Teqing positive lines were much higher than pRi-*BOC1*-YIL25 positive transgenic lines and pCPL-*BOC1*-Teqing positive transgenic lines, and it was not easy to find the line to line variations. Therefore, we made three figures with the expression of *BOC1* and CBI, respectively (**Fig. 2**). Meanwhile, we added this result to our revised manuscript (**Fig. 3**, line125-127, 133-134,142-144).

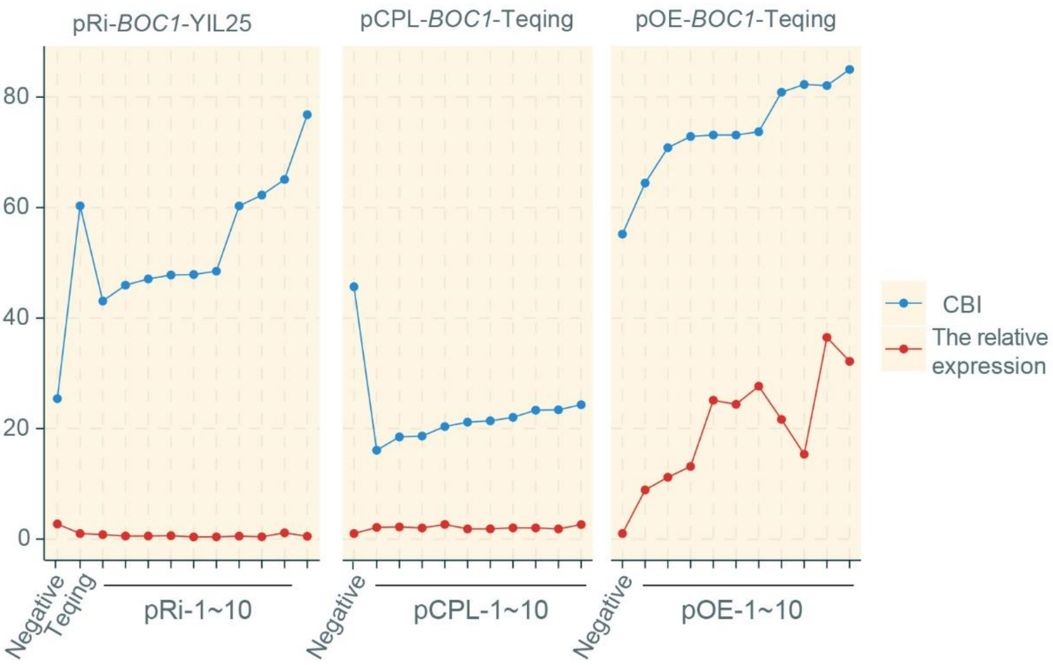

Fig. 1 The line to line variations with pRi-*BOC1*-YIL25, pCPL-*BOC1*-Teqing and pOE-*BOC1*-Teqing.

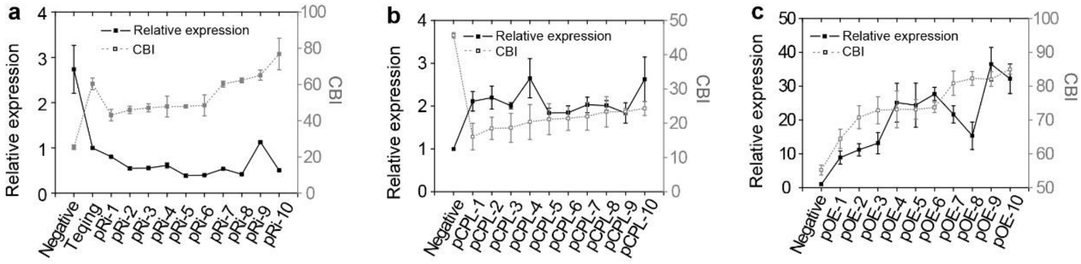

Fig. 2 The line to line variations with pRi-*BOC1*-YIL25, pCPL-*BOC1*-Teqing and pOE-*BOC1*-Teqing.

(2) Yes, actually, our previous differential expression analysis was insufficient and deficient. In our previous study, only differential expression of Teqing and YIL25 was analyzed. However, YIL25 contained three YJCWR chromosomal segments on the short arm of chromosomes 2, 3, and 5 and other genes were also present in the same YJCWR chromosomal segments, which affected the accuracy of the results. Following your suggestion, in our revised manuscript, we added RNA-seq experiments using callus that had been subcultured for 21 days from Teqing and the complementation line pCPL-*BOC1*-Teqing (pCPL), and Teqing and the overexpression line pOE-*BOC1*-Teqing (pOE). We identified a total of 700 differentially expressed genes (DEGs), as well as 410 DEGs upregulated in the callus of YIL25 versus Teqing, pCPL versus Teqing and downregulated in pOE versus Teqing (UUD, **Fig. 7a**, **Supplementary**

Table. 2). Besides, 290 DEGs downregulated in the callus of YIL25 versus Teqing and pCPL versus Teqing and upregulated in pOE versus Teqing (DDU, fold change ≥ 2, FDR < 0.001) (**Fig. 7b**, **Supplementary Table. 3**). A large number of differentially expressed genes related to ROS production, stress response and cell death were found in the overlap of UUD or DDU. These results supported that appropriate improving expression level of *BOC1* were beneficial for the cells to be in redox state, prevented various stress and then inhibited cell death, while the overexpression lines on the contrary. These results were described in the revised manuscript (line 233-275).

R3Q2: A description of the experiment that resulted in the data for fig. 5 is missing in the material and methods. It is not clear how many calli were used for transformation in each experiment. Three experiments (for data in fig. 5) seems a small number considering that typically large variation is found in transformation efficiency from experiment to experiment. Therefore, at least 10 independent experiments should be presented for figure 5.

Response: Thank you very much for your valuable comments.

According to your suggestions, we performed additional 7 independent experiments and also exhibited the *Hyg*-resistant calli of the 10 independent experiments with Teqing 1300, YIL25 1300 and Teqing pCPL, respectively (**Fig. 1a-c**). The result indicated that the selection frequency of *Hyg*-resistant calli in YIL25 1300 was more than 2.7-fold higher than that in Teqing 1300 **(Fig. 5a-b, d)**, and the transformation efficiency in YIL25 1300 was 2.5-fold higher than in the Teqing 1300 control **(Fig. 5e)**, which indicated that YIL25-containing *BOC1* was an excellent receptor material for genetic transformation. Meanwhile, the selection frequency of *Hyg*-resistant calli in Teqing pCPL was improved 2.1 times **(Fig. 5a, c, d)**, and the transformation efficiency was remarkably improved 1.9 times compared to the Teqing 1300 control **(Fig. 5e)**. This is more powerful proof that *BOC1* improved selection frequency of *Hyg*-resistant calli and genetic transformation efficiency by reducing callus browning in the process of *Agrobacterium*-mediated transformation.

Meanwhile, we added a description of the number of calli which used for transformation in each experiment ("The means represent data from 10 groups of independent experiments and the number of transformed callus in each group was ~80-120") in the method of "*Agrobacterium*-mediated genetic transformation" (line 479-481).

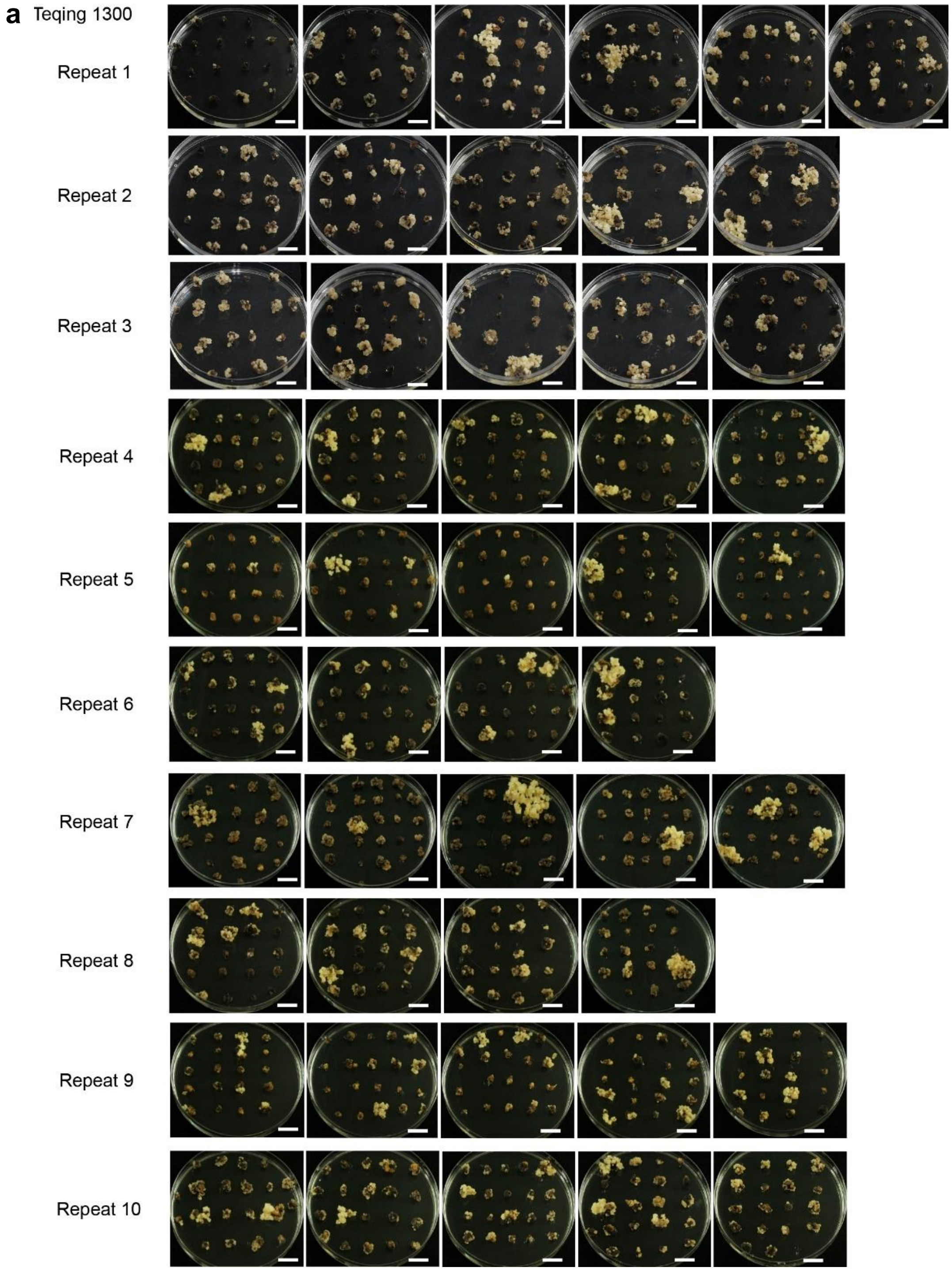

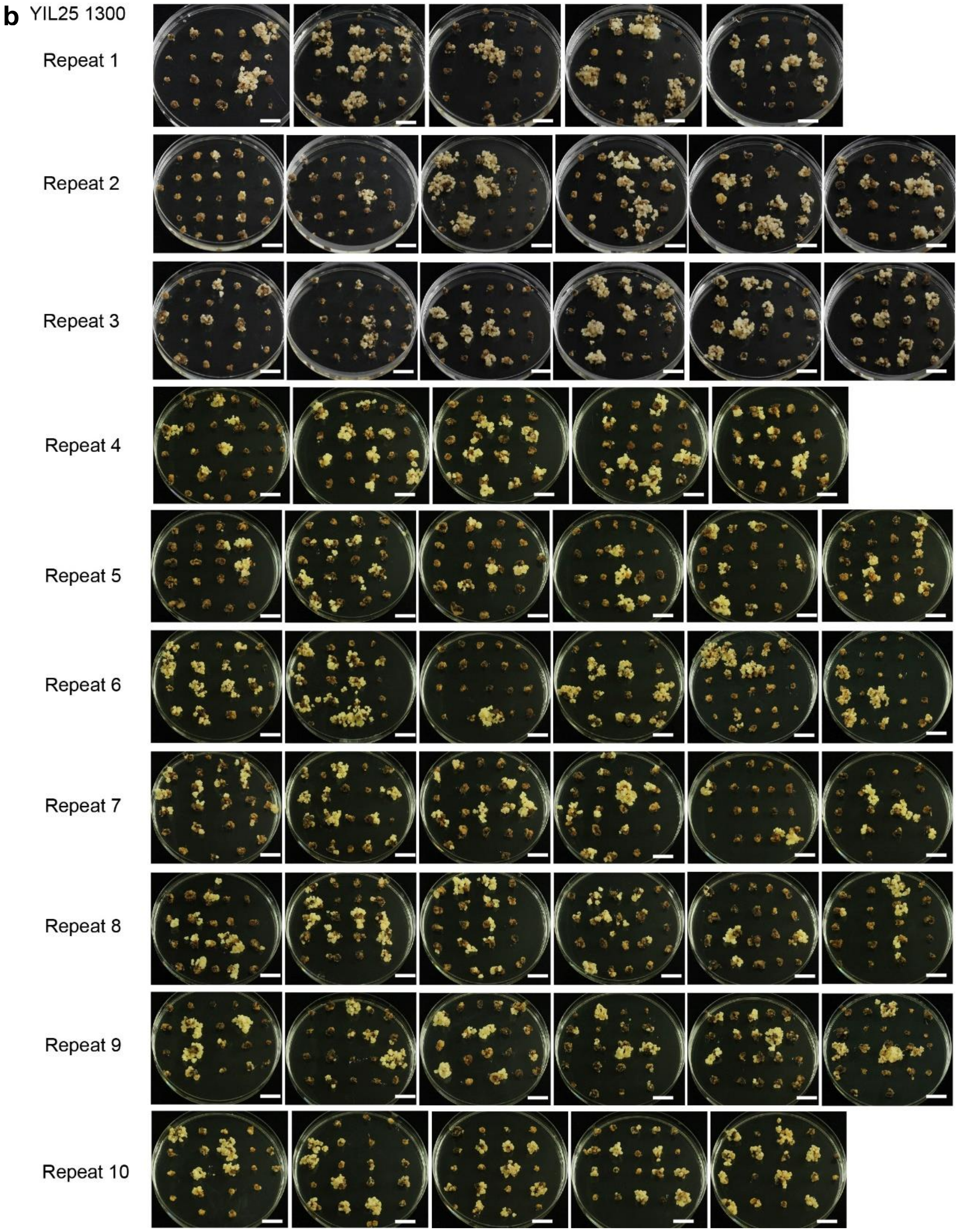
b
YIL25 1300
Repeat 1
Repeat 2
Repeat 3
Repeat 4
Repeat 5
Repeat 6
Repeat 7
Repeat 8
Repeat 9
Repeat 10

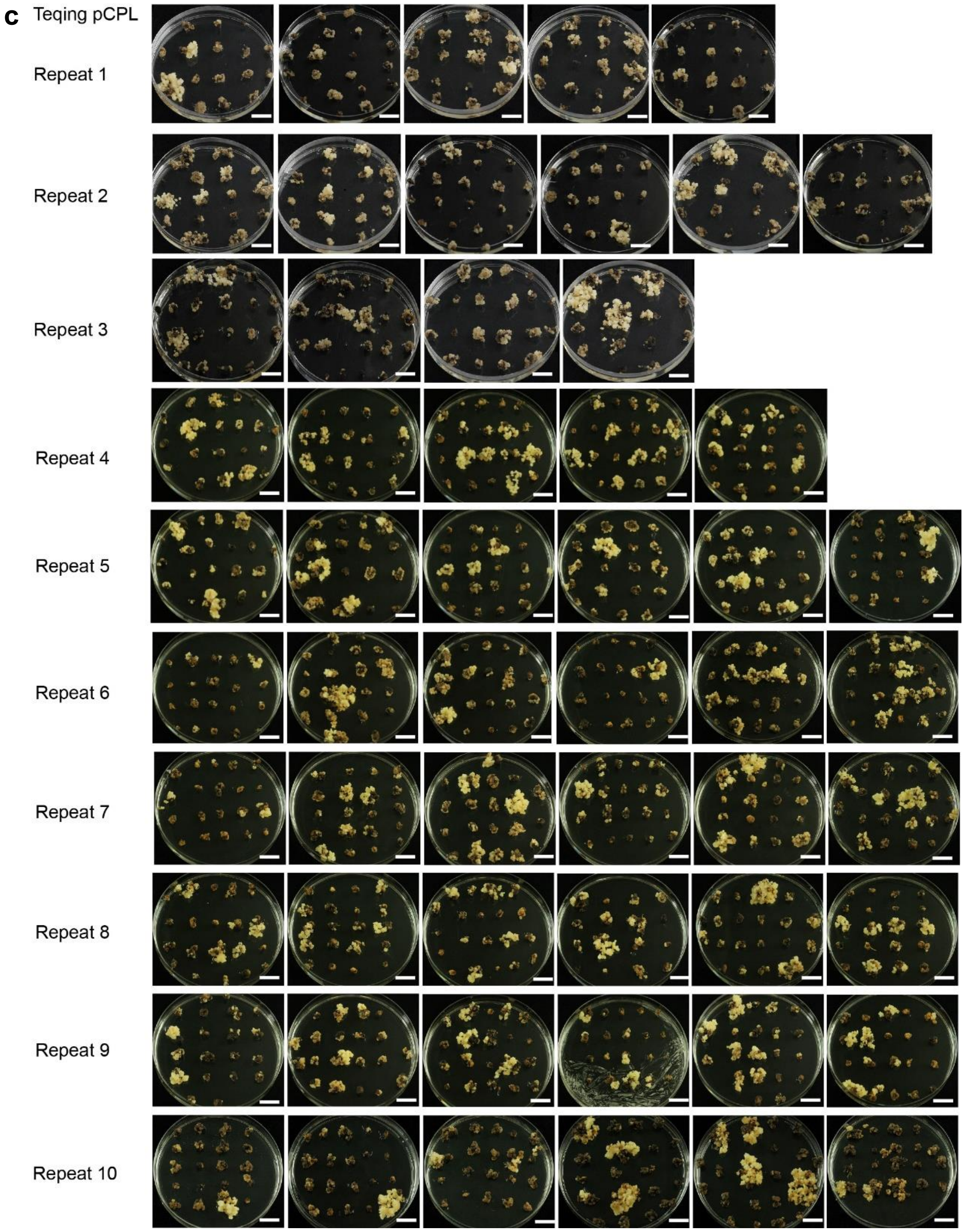

Fig. 1 The *Hyg*-resistant calli of Teqing 1300,YIL25 1300 and Teqing pCPL.

Scale bars, 1 cm.

Minor aspects that can be addressed to improve the manuscript:

Results:

Line 90: Rephrase “the cells of Teqing appeared introcession”

Line 94-95: “First, we measured the CBI on the seeds of the 198 individual

plants.” Please clarify if the CBI was assessed on the seeds or on seed derived callus. Also include data in supplementary file or cite paper if published elsewhere.

Line 123-124: Replace “containing the promoter and open reading frame of LOC_Os03g12820” with “containing the promoter, open reading frame and 3’ UTR of LOC_Os03g12820”

Line 130: Rephrase to “results suggest that LOC_Oso3g12820 is BOC1 regulates callus browning in rice.”

Line 153-154: Rephrase to “ which indicated that the expression of BOC1 in YIL25 calli was higher than in Teqing calli (Fig. 4b).”

Line 180: Rephrase to “lines were worse”

Response: Thank you for your helpful comments.

(1) Following your suggestion, we modified the “the cells of Teqing appeared introcession ” to “the cells of Teqing were sunk” (line 92).

(2) The CBI was assessed on the seeds derived callus. We added this to our modified manuscript of line 96. Moreover, we added the QTL analysis **(Supplementary Table. 1)** and the phenotype and genotype data of 198 individual plants **(Source Data file of Fig. 2a)** to our revised manuscript.

(3) Following your comment, we modified “containing the promoter and open reading frame of *LOC_Os03g12820*” to ”containing the promoter, open reading frame and 3′-UTR of *LOC_Os03g12820*” (line 128-129).

(4) We revised “these results suggest that *LOC_Oso3g12820* is *BOC1*, which regulates callus browning in rice” to “these results suggest that *LOC_Oso3g12820* is *BOC1* regulates callus browning in rice” according to your suggestion (line135-136).

(5) Following your suggestion, we modified the sentence “which showed the expression of *BOC1* in YIL25 calli was higher than that in Teqing calli (Fig. 4b)”

to “which indicated that the expression of *BOC1* in YIL25 calli was higher than in Teqing calli (Fig. 4b) ” (line 168-169).

(6) According your suggestion, we modified the “lines showed worse” to “lines were worse” (line 142).

Material and Methods:

Line 332-333. Transformation efficiency is defined as “(number of positive plants for independent transgenic events/number of transferred calli) ×100%” and should be: “(number of independent transgenic events regenerating plants/number of transferred calli) ×100%”

Line 684: Figure legend for Fig. 5 “Data were repeated three times” Do the authors intend to state “means represent data from three independent experiments”?

Figure legends for Figure 3 and supplementary figure 7: For clarity the genetic background of the “negative transgenic line” should be described.

Response: Thank you very much for your positive suggestions.

(1) According to your suggestion, we revised this formula to “Transformation efficiency = (number of independent transgenic events regenerating plants /number of transferred calli) ×100%” (line 410).

(2)Yes, we intend to state “means represent data from three groups of independent experiments”. Meanwhile, combining your first comment, we revised this sentence of line 479-481 to “The means in the test of the efficiency of genetic transformation represent data from 10 groups of independent experiments and the number of transformed callus in each group was ~80-120”.

(3) The “negative” refers to the segregating plants from transformation process but without the transgene. In response to your suggestion, we had added the description of “negative transgenic line” in the Figure 3 legend of our modified manuscript (line 770-771).

REVIEWERS' COMMENTS:

Reviewer #1 (Remarks to the Author):

The authors addressed my comments and I only have some minor suggestions:
1. Row 31 – 33 "The presence of the Tourist-like miniature inverted-repeat transposable element (Tourist MITE) specific to wild rice in the promoter of BOC1 increases the expression of BOC1 in callus."
This is the first time you introduce the MITE so you should use "a" instead of "the":
"The presence of a Tourist-like miniature inverted-repeat transposable element (Tourist MITE) specific to wild rice in the promoter of BOC1 elevates the expression of BOC1 in callus."
2. Row 308 – 309 "we further founded" should be "we further found".
3. Row 806 "efficiency of 10 repeats" should be "efficiency of 10 replicates".

Reviewer #2 unavailable.

Reviewer #3 (Remarks to the Author):

The reviewers' suggestions were implemented and improved the quality of the manuscript. Language needs further editing and the authors are advised to hire a reputed professional editing service. Some suggested changes are included below. Important for the reproducibility of the study is to present the sequence of the over-expression construct more clearly: Line 129 include the nucleotide sequence of the introduced construct in the supplementary file or refer to supplementary figure 3 and indicate that the 4752 nt sequence shown for the entire expression cassette of BOC1 from YIL25 was used for construction of the overexpression construct (if this is what was used) as shown in supplementary figure 3.

Minor changes:
Line 57 to 59 change to: However, to our knowledge, there have been no reports on the isolation of genes related to callus browning in any plant species. The molecular mechanisms underlying physiological processes inducing browning in plant cell culture still remain unclear.
Line 68 -79 change to: BOC1 improved the genetic transformation efficiency and frequency of Hygromycin (Hyg)-resistant calli following gene transfer.

Line 90-92 change to: Observations using scanning electron microscopy (SEM) revealed that YIL25 calli consisted of many globular nodules (Fig. 1h) and the cells of YIL25 were turgid (Fig. 1i), whereas Teqing calli had fewer globular nodules (Fig. 1f) and the cells of Teqing were flaccid (Fig. 1g).

Line 97 change to: First, we measured the CBI on the seed derived callus of
the 198 individual plants.

Line 124 to 127 change to: In addition, RNAi suppression of LOC_Os03g12820 in the pRi-BOC1-YIL25 positive transgenic lines to the level of Teqing caused serious callus browning compared to the non-transformed controls (Fig. 3a-e).

Line 130-134 change to: RT-qPCR analysis showed that the relative expression levels of LOC_Os03g12820 was significantly elevated in the pCPL-BOC1-Teqing positive plants compared to the negative plants (Teqing) (Fig. 3j). The CBI was reduced in these positive lines when the expression level of LOC_Os03g12820 was elevated at least 1.8 fold compared with the negative plants (Fig. 3f-j).

Line 304 to 313

To ascertain whether BOC1 was responsible for callus browning in Asian rice cultivars, firstly, an association analysis was carried out between callus browning and sequence variations of 74 Asian rice cultivars. The result indicated that the Indel8 (-433, T indel) in the promoter of BOC1 was the strongest signal. However, this site was not polymorphic in the 50 accessions of O. rufipogon (Fig. 8d; Supplementary Table. 4, 5). We further found that the CBI in 15 varieties with T insertion in the promoter of BOC1 was lower than that in 59 varieties without this insertion (Fig. 8e; Supplementary Table. 5). We also analyzed the expression levels in these varieties and the result showed that the expression levels of BOC1 was higher in the 15 varieties with T insertion in the promoter of BOC1 than that in 59 varieties without this insertion (Fig. 8f;Supplementary Table 5).The above results suggest that the sequence variation in the BOC1 promoter influence callus browning by affecting the expression level of BOC1.
Line 361 to 363 change to: The RNA-seq data analysis revealed that BOC1 influences the expression of genes associated with ROS production and scavenging, stress response and cell death.

Reviewer #4 (Remarks to the Author):

All rice SROs have a group I SRO (WWE-PARP-RST) domain architecture similar to Arabidopsis RCD1 and SRO1. Indeed, monocots and Physcomitrella lack the Group IIa and IIb SROs, which have a PARP-RST domain architecture and are found in all other plants. Remarkably, the PARP domains of other rice SROs (OsSRO1a and OsSRO1b) have a higher level of similarity to RCD1 and SRO1 (Jaspers et al. BMC Genomics 2010) than does OsSRO1c. OsSRO1c belongs to the group Ic clade of SROs that does not have representatives in Arabidopsis. Furthermore, another paper shows OsSRO1c to be more similar to the clade containing AtSRO3-AtSRO5, than it is to the clade containing RCD1 and SRO1 (see Fig 2b, You et al., JExBot 2016), although the methods used to create this phylogeny are not clearly stated. Nonetheless, care must be taken to not draw too close a comparison between OsSRO1c and RCD1, which, as would be expected given their evolutionary histories, have contrasting functions in the regulation of ROS related processes.

The phylogeny in figure S4b only has RCD1 from A. thaliana (CEO1 is synonymous to RCD1), RCD1 from A. lyrata, a WWE protein family from A. thaliana (which must be either RCD1 or SRO1), and then many SROs from plants in the Gramineae. This figure might inadvertently exaggerate the similarity of OsSRO1c to RCD1 and SRO1.

The comparison and contrasts to RCD1 are warranted but perhaps a few sentences could be added to clarify the relationship of OsSRO1c to the members of the Arabidopsis RCD1/SRO gene family. This would make clear that while conserved mechanisms may govern RCD1 and OsSRO1c function, it is not surprising, and even to be expected, that these two proteins regulate several ROS signaling related processes in a completely opposite manner. Addition of other Arabidopsis SROs to Figure S4b would also help illuminate these relationships, although I don’t see modifying this figure as a requirement for publication.

It may also be good if a little discussion were devoted to RCD1 function as there are recently published advances.

Kirk Overmyer

[Editor: Reviewer #4 states in Remark to Editor section that (s)he thinks previous Reviewer #2’s suggestions have been adequately addressed. However, Reviewer #4 asks to carefully state the conclusion on BOC1 preventing callus browning via the inhibition of cell death as cell death was not

directly measured. In addition, in regard to the recently published advances on RCD1 function, Reviewer #4 refers to Shapiguzov et al 2019, eLife.]

Dear Reviewers,

Thank you very much for your excellent comments on our manuscript "**A common wild rice-derived *BOC1* allele reduces callus browning in *indica* rice transformation**" (**ID: NCOMMS-19-09797**). We have studied comments carefully and have revised the manuscript. We used red color to mark the modified words and sentences in main text and figure legends. The detailed responses are shown below. We would be grateful if you can kindly be satisfied with the revised manuscript.

Thank you again for your time and efforts in taking care of our manuscript.

Sincerely,

Yongcai Fu, Ph.D., Professor
Department of Plant Genetics and Breeding
China Agricultural University, Beijing 100193, China
Tel: 86-10-62733507
E-mail: yongcaifu@cau.edu.cn

Reviewers' comments:

Reviewer #1 (Remarks to the Author):

The authors addressed my comments and I only have some minor suggestions:

Response: Thank you very much for your positive comments.

1. Row 31 – 33 “The presence of the Tourist-like miniature inverted-repeat transposable element (Tourist MITE) specific to wild rice in the promoter of BOC1 increases the expression of BOC1 in callus.”

This is the first time you introduce the MITE so you should use “a” instead of “the”: “The presence of a Tourist-like miniature inverted-repeat transposable element (Tourist MITE) specific to wild rice in the promoter of BOC1 elevates the expression of BOC1 in callus.”

Response: Following your suggestion, we have modified this word of the sentence in line 33-35.

2. Row 308 – 309 “we further founded” should be “we further found”.

Response: We have revised this error as your suggest (line 352-354).

3. Row 806 “efficiency of 10 repeats” should be “efficiency of 10 replicates”.

Response: We have modified this word (line 949).

Reviewer #2 unavailable.

Reviewer #3 (Remarks to the Author):

The reviewers’ suggestions were implemented and improved the quality of the manuscript.

Language needs further editing and the authors are advised to hire a reputed professional editing service. Some suggested changes are included below. Important for the reproducibility of the study is to present the sequence of the over-expression construct more clearly: Line 129 include the nucleotide sequence of the introduced construct in the supplementary file or refer to supplementary figure 3 and indicate that the 4752 nt sequence shown for the entire expression cassette of BOC1 from

YIL25 was used for construction of the overexpression construct (if this is what was used) as shown in supplementary figure 3.

Response: Thank you very much for your helpful suggestions.

(1) We thoroughly edited English grammar and usage through a professional editing service: Plant Biology Science Editors (https://planteditors.com).

(2) We have added the description of the sequence of the over-expression construct "as shown in Supplementary Fig. 3, the rose red and red underline represent the nucleotide sequence of the overexpression construct" (line 151-152).

Minor changes:

Line 57 to 59 change to: However, to our knowledge, there have been no reports on the isolation of genes related to callus browning in any plant species. The molecular mechanisms underlying physiological processes inducing browning in plant cell culture still remain unclear.

Response: We have modified it as your comment (line 61-64).

Line 68 -79 change to: BOC1 improved the genetic transformation efficiency and frequency of Hygromycin (Hyg)-resistant calli following gene transfer.

Response: We have modified it accordingly (line 73-75).

Line 90-92 change to: Observations using scanning electron microscopy (SEM) revealed that YIL25 calli consisted of many globular nodules (Fig. 1h) and the cells of YIL25 were turgid (Fig. 1i), whereas Teqing calli had fewer globular nodules (Fig. 1f) and the cells of Teqing were flaccid (Fig. 1g).

Response: We have revised it as your suggestion (line 97-101).

Line 97 change to: First, we measured the CBI on the seed derived callus of the 198 individual plants.

Response: As your comment, we have modified it (line 106-107).

Line 124 to 127 change to: In addition, RNAi suppression of LOC_Os03g12820 in the pRi-BOC1-YIL25 positive transgenic lines to the level of Teqing caused serious callus browning compared to the non-transformed controls (Fig. 3a-e).

Response: We have modified it as your suggestion (line 135-139).

Line 130-134 change to: RT-qPCR analysis showed that the relative expression levels of LOC_Os03g12820 was significantly elevated in the pCPL-BOC1-Teqing positive plants compared to the negative plants (Teqing) (Fig. 3j). The CBI was reduced in these positive lines when the expression level of LOC_Os03g12820 was elevated at least 1.8 fold compared with the negative plants (Fig. 3f-j).

Response: We have modified it as your comment (line 142-147).

Line 304 to 313

To ascertain whether BOC1 was responsible for callus browning in Asian rice cultivars, firstly, an association analysis was carried out between callus browning and sequence variations of 74 Asian rice cultivars. The result indicated that the Indel8 (-433, T indel) in the promoter of BOC1 was the strongest signal. However, this site was not polymorphic in the 50 accessions of O. rufipogon (Fig. 8d; Supplementary Table. 4, 5). We further found that the CBI in 15 varieties with T insertion in the promoter of BOC1 was lower than that in 59 varieties without this insertion (Fig. 8e; Supplementary Table. 5). We also analyzed the expression levels in these varieties and the result showed that the expression levels of BOC1 was higher in the 15 varieties with T insertion in the promoter of BOC1 than that in 59 varieties without this insertion (Fig. 8f;Supplementary Table 5).The above results suggest that the sequence variation in the BOC1 promoter influence callus browning by affecting the expression level of BOC1.

Response: We have modified it according your suggestion (line 347-360), thanks a lot.

Line 361 to 363 change to: The RNA-seq data analysis revealed that BOC1 influences the expression of genes associated with ROS production and scavenging, stress response and cell death.

Response: We have modified it in our manuscript of revision (line 412-415).

Reviewer #4 (Remarks to the Author):

All rice SROs have a group I SRO (WWE-PARP-RST) domain architecture similar to Arabidopsis RCD1 and SRO1. Indeed, monocots and Physcomitrella lack the Group

IIa and IIb SROs, which have a PARP-RST domain architecture and are found in all other plants. Remarkably, the PARP domains of other rice SROs (OsSRO1a and OsSRO1b) have a higher level of similarity to RCD1 and SRO1 (Jaspers et al. BMC Genomics 2010) than does OsSRO1c. OsSRO1c belongs to the group Ic clade of SROs that does not have representatives in Arabidopsis. Furthermore, another paper shows OsSRO1c to be more similar to the clade containing AtSRO3-AtSRO5, than it is to the clade containing RCD1 and SRO1 (see Fig 2b, You et al., JExBot 2016), although the methods used to create this phylogeny are not clearly stated. Nonetheless, care must be taken to not draw too close a comparison between OsSRO1c and RCD1, which, as would be expected given their evolutionary histories, have contrasting functions in the regulation of ROS related processes.

Response: Thank you for your helpful comment. According your suggestion, we remove the sentence "Arabidopsis plants overexpressing *RCD1* are more susceptible to ozone treatment than the wild type, but less so than *rcd1* mutants, which indicates that an optimum expression level of *RCD1* contributes to the proper function[44]"(line 379-382).

The phylogeny in figure S4b only has RCD1 from A. thaliana (CEO1 is synonymous to RCD1), RCD1 from A. lyrata, a WWE protein family from A. thaliana (which must be either RCD1 or SRO1), and then many SROs from plants in the Gramineae. This figure might inadvertently exaggerate the similarity of OsSRO1c to RCD1 and SRO1.

The comparison and contrasts to RCD1 are warranted but perhaps a few sentences could be added to clarify the relationship of OsSRO1c to the members of the Arabidopsis RCD1/SRO gene family. This would make clear that while conserved mechanisms may govern RCD1 and OsSRO1c function, it is not surprising, and even to be expected, that these two proteins regulate several ROS signaling related processes in a completely opposite manner. Addition of other Arabidopsis SROs to Figure S4b would also help illuminate these relationships, although I don't see modifying this figure as a requirement for publication.

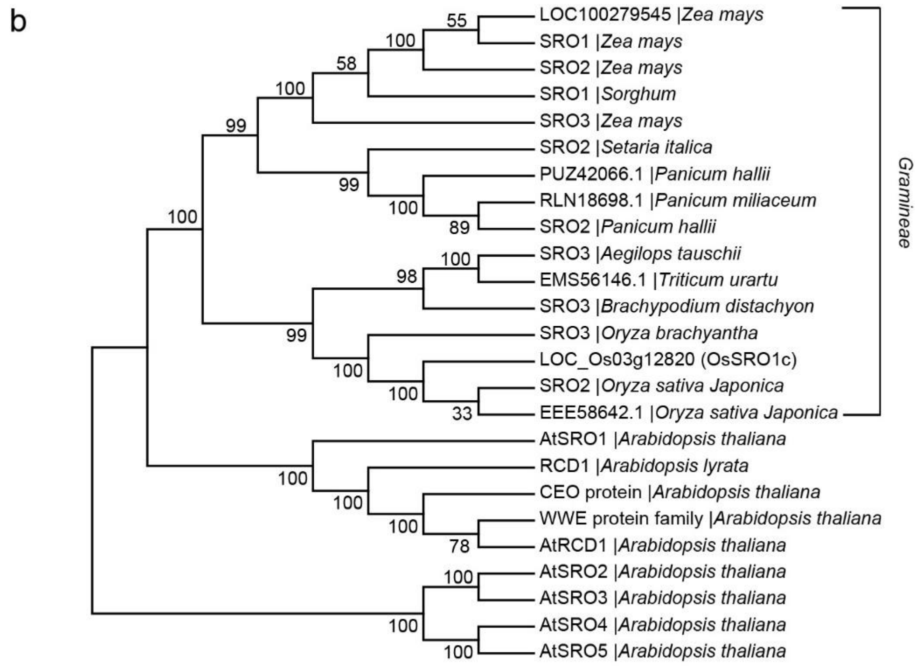

Supplementary Fig. 4b Phylogenetic analysis (b) of BOC1 protein.

Response: Thank you for your suggestion. According your comment, we have added other *Arabidopsis* SROs to Figure S4b, and added the sentence “However, as shown in Supplementary Fig. 4b, RCD1 and OsSRO1c have different evolutionary histories which may have contrasting functions in the regulation of ROS related processes” in our revision (line 419-421).

It may also be good if a little discussion were devoted to RCD1 function as there are recently published advances.

Kirk Overmyer

[Editor: Reviewer #4 states in Remark to Editor section that (s)he thinks previous Reviewer #2’s suggestions have been adequately addressed. However, Reviewer #4 asks to carefully state the conclusion on BOC1 preventing callus browning via the inhibition of cell death as cell death was not directly measured. In addition, in regard to the recently published advances on RCD1 function, Reviewer #4 refers to

Shapiguzov et al 2019, eLife.]

Response: Thank you for your helpful and valuable comments.

(1)Yes, we could not state the conclusion *BOC1* prevented callus browning via the inhibition of cell death as cell death was not directly measured. However, the results of assays of physiological indices and plant hormones related to oxidative stress and stress responses genes of RNA-seq data showed indirectly that *BOC1* preventing callus browning may be through inhibition of cell death. As your suggestion, we modified the sentence of line 233-234, 258-259, and 311-313. We have removed the sentence of “Therefore, our findings demonstrated that callus browning is caused by PCD due to oxidative stress” (line 415-416).

(2) According your suggestion, we have added the sentences “In recently report, RCD1 coordinates chloroplast and mitochondrial functions through interaction with transcription factors. RCD1 participates in regulation of mitochondrial respiration and chloroplast ROS processing and may contribute to the survival of plant under a changing environment[55]” (line 416-419) and “In the current study, we use the calli subcultured for 21 d in the dark and is not necessary for photosynthesis by origin of carbon from medium. Therefore, the interaction and phenotype of BOC1 may be different from seedling stage, which will be an interesting research topic” (line 421-424).

55. Shapiguzov, A. et al. Arabidopsis RCD1 coordinates chloroplast and mitochondrial functions through interaction with ANAC transcription factors. *eLife* **8**: e43284 (2019).
